# Induction of IL-10 and TGFβ from CD4^+^CD25^+^FoxP3^+^ T Cells Correlates with Parasite Load in Indian Kala-azar Patients Infected with *Leishmania donovani*

**DOI:** 10.1371/journal.pntd.0004422

**Published:** 2016-02-01

**Authors:** Pradyot Bhattacharya, Smriti Ghosh, Sarfaraz Ahmad Ejazi, Mehebubar Rahaman, Krishna Pandey, Vidya Nand Ravi Das, Pradeep Das, Rama Prosad Goswami, Bibhuti Saha, Nahid Ali

**Affiliations:** 1 Infectious Diseases and Immunology Division, Indian Institute of Chemical Biology, Kolkata, West Bengal, India; 2 Department of Tropical Medicine, School of Tropical Medicine, Kolkata, West Bengal, India; 3 Department of Clinical Medicine, Rajendra Memorial Research Institute of Medical Sciences, Patna, Bihar, India; 4 Department of Molecular Biology, Rajendra Memorial Research Institute of Medical Sciences, Patna, Bihar, India; Lancaster University, UNITED KINGDOM

## Abstract

**Background:**

Visceral leishmaniasis (VL) is distinguished by a complex interplay of immune response and parasite multiplication inside host cells. However, the direct association between different immunological correlates and parasite numbers remains largely unknown.

**Methodology/Principal Findings:**

We examined the plasma levels of different disease promoting/protective as well as Th17 cytokines and found IL-10, TGFβ and IL-17 to be significantly correlated with parasite load in VL patients (r = 0.52, 0.53 and 0.51 for IL-10, TGFβ and IL-17, respectively). We then extended our investigation to a more antigen-specific response and found leishmanial antigen stimulated levels of both IL-10 and TGFβ to be significantly associated with parasite load (r = 0.71 and 0.72 for IL-10 and TGFβ respectively). In addition to cytokines we also looked for different cellular subtypes that could contribute to cytokine secretion and parasite persistence. Our observations manifested an association between different Treg cell markers and disease progression as absolute numbers of CD4^+^CD25^+^ (r = 0.55), CD4^+^CD25^hi^ (r = 0.61) as well as percentages of CD4^+^CD25^+^FoxP3^+^ T cells (r = 0.68) all correlated with parasite load. Encouraged by these results, we investigated a link between these immunological components and interestingly found both CD4^+^CD25^+^ and CD4^+^CD25^+^FoxP3^+^ Treg cells to secrete significantly (*p*<0.05) higher amounts of not only IL-10 but also TGFβ in comparison to corresponding CD25^-^ T cells.

**Conclusions/Significance:**

Our findings shed some light on source(s) of TGFβ and suggest an association between these disease promoting cytokines and Treg cells with parasite load during active disease. Moreover, the direct evidence of CD4^+^CD25^+^FoxP3^+^ Treg cells as a source of IL-10 and TGFβ during active VL could open new avenues for immunotherapy towards cure of this potentially fatal disease.

## Author Summary

Visceral leishmaniasis (VL) is one of the most widespread parasitic diseases worldwide and is caused by kinetoplastid protozoa of the *Leishmania donovani* complex. The disease begins with internalization of *L*. *donovani* parasites and their multiplication within host macrophages followed subsequently by immune suppression. However, the immunological factors responsible for disease progression and their association with parasite dynamics are not completely understood. Herein, we investigated the correlation of different immune components (cytokines and cellular subsets) with parasite load and their involvement in the course of VL. Our study revealed a significant positive correlation between parasite load and plasma as well as antigen specific levels of IL-10 and TGFβ. In addition to cytokines, cellular subsets could also contribute to disease pathogenesis through their regulatory mechanisms. Our results indicate different Treg cell markers (absolute numbers of CD4^+^CD25^+^ and CD4^+^CD25^hi^ and percentages of CD4^+^CD25^+^FoxP3^+^) to be strongly correlated with parasite load. Exploring an association between these immunological correlates revealed Treg cells to be the source of these cytokines during VL. Therefore, this study points to a significant role of IL-10, TGFβ and Treg cells in parasite load and active VL, providing evidence which could be helpful in devising new immunotherapeutic strategies against this disease.

## Introduction

Visceral leishmaniasis (VL) or kala-azar, a deadly infection affecting the organs of lymphoreticular system, is caused by the protozoan parasites of the *Leishmania donovani* complex. Human infections can be asymptomatic or oligosymptomatic with manifestations including persistent low grade fever, weight loss, hepatosplenomegaly, lymphadenopathy and cachexia. Other disease specific characteristics include pancytopenia, hypergammaglobulinemia, hypoalbuminemia and severe parasite infestations in visceral organs such as the liver and spleen, and in the bone marrow [1]. An estimate of 0.2–0.4 million global VL cases are reported each year with more than 90% of them occurring in India, Bangladesh, Sudan, South Sudan, Brazil and Ethiopia [2]. In India, the disease is mostly prevalent in the state of Bihar, parts of Eastern Uttar Pradesh and West Bengal [3] with an estimated annual incidence (cases from 2004–2008) ranging from 146,700 to 282,000 and a mortality rate of 2.4% (853/34,918) [2].

The mechanisms underlying the multiplication and spread of parasites in VL are not well understood. Infection with *Leishmania* parasites does not always lead to illness and the majority of people residing in endemic regions never develop VL. How these individuals develop resistance against infection is an area of intense interest but remains largely unknown. In most tegumentary forms of leishmaniasis parasite growth is restricted and antileishmanial cell mediated immunity (CMI) is developed [4]. However, the absence of antigen specific CMI has been regarded as a signature of VL [5]. Development of a Th1 type immunity appears to be needed for limiting parasite growth and resistance against infection [6]. PBMCs from clinically asymptomatic subjects have been observed to respond to triggering with leishmanial antigen and secrete IL-2, IFNγ and IL-12 [7]. However, a key immunological attribute of VL remained is the apparent incapability of its PBMCs to manifest an antigen specific immune response as suggested by their failed proliferative [8, 9] or cytokine producing abilities [10, 11]. Although active VL was initially characterized as presenting a predominant Th2 type response with elevated levels of serum IL-4 and IL-13 [12, 13], other studies reported increased levels of IFNγ and IL-12 along with IL-5 and IL-6 in plasma [14–17] as well as enhanced IFNγ mRNA expression [11, 18, 19] during disease, suggesting that its development is not due to a predominant Th2 response. Rather clinical studies implicate a strong role for IL-10 associated with the immunosuppressive nature of kala-azar [10]. VL patients have been noted for an augmented production/expression of IL-10 at both cellular [15–17, 20] and molecular levels [18, 19, 21]. More recently IL-10 could be measured in leishmanial antigen-stimulated PBMC from culture supernatants [17, 20, 22] and whole blood cultures of VL patients [23, 24]. Another cytokine important from the perspective of human VL pathology is TGFβ [25, 26]. Its elevation has been reported in plasma as well as antigen-stimulated PBMCs of VL patients [20, 22].

IL-10 regulated parasite progression and development of infection have been linked with accumulation of CD4^+^CD25^+^ regulatory T cells (Treg) in both experimental [27] and human cutaneous leishmaniasis (CL) [28]. Natural Treg cells were found to secrete large amounts of IL-10 and/or TGFβ in CL patients [28, 29] and asymptomatic *L*. *major* infected individuals [30], as well as in PBMCs of healthy subjects activated by live *L*. *guyanensis* parasites [31]. However, although CD4^+^CD25^+^ T cells have been related with kala-azar [20] and patients suffering from PKDL [20], FoxP3^-^CD4^+^ T cells were reported to be the key source of enhanced IL-10 mRNA expression in spleen of VL patients [11]. Still other reports indicate that CD4^+^CD25^+^FoxP3^+^ cells could play a part in human VL as a vital source of IL-10 [32]. Th17 cells, an independently regulated and highly proinflammatory subset of CD4^+^ T cells, are also known to produce IL-17 and IL-22 [33], effectors of innate immunity. Activation of Th17 cells is characterized by neutrophil recruitment and depending on their differentiation program they can either cause damaging effects or play a role in protection [34]. While Th17 cells have been associated with pathological roles and tissue damage in human mucocutaneous leishmaniasis [35], a protective role has been attributed to them in the case of human VL [36]. CD8^+^ T cells, by virtue of their cytotoxic activity, lyse pathogen-infected cells and produce IFNγ thereby conferring protection to the host. However, they can also act as suppressor T cells, down-modulating the host immune response and thereby increasing the chances of survival of invading parasites inside their hosts [37]. Thus CD8 T cells have the potential to contribute to both resolution and pathogenesis of VL. Interestingly a low CD4 to CD8 ratio has been reported in PBMC from VL patients [38]. However, the mechanisms of evasion of CD8^+^ T cell-mediated host protective immunity by *L*. *donovani* in human VL are largely unknown.

To understand how the immune response generated during VL in the host is impacted by the multiplication of the pathogen resulting in clinical pathogenesis and disease, we have studied the direct relation between different immunological correlates and the number of parasites in VL patients. Our study demonstrates, in addition to IL-10, for the first time a positive correlation of TGFβ in plasma, as well as IL-10 and TGFβ in antigen-stimulated PBMCs from culture supernatants, with parasite loads of VL patients, supporting their roles in disease pathology. Additionally, our results also point to a positive correlation between absolute numbers of CD4^+^CD25^+^ Treg cells with parasite load, highlighting their involvement in *L*. *donovani* infection. On the basis of the initial results we further investigated whether these cellular subsets could be the sources of disease promoting cytokines (IL-10 and TGFβ), and our observations revealed, along with IL-10, for the first time TGFβ to be secreted from CD4^+^CD25^+^FoxP3^+^ Treg cells during active VL.

## Materials and Methods

### Healthy controls and patients

Twenty VL patients (males and females, and HIV negative) admitted to School of Tropical Medicine (STM), Kolkata, West Bengal, and Rajendra Memorial Research Institute of Medical Sciences (RMRIMS), Patna, Bihar were included in this study (Table 1). Patients were initially diagnosed by the usual clinical presentations such as prolonged fever, hepatosplenomegaly and pancytopenia. Confirmation criteria for the disease, however, was restricted to the rK39 strip test, as the risky and painful nature of aspiration (both splenic and bone marrow) techniques limit them to be employed for all patients studied. 4–5 ml of venous blood samples were collected from all patients. The patients were then subjected to a standard treatment protocol as selected by the study centers (STM and RMRIMS) and discharged after completion of therapy as clinically cured. The non-endemic healthy individuals (NEC), both male and female, included in the study were from different areas of Kolkata with an age range of 23–35 years. Endemic healthy individuals (EC) [healthy family members living in the same endemic regions of Bihar] were chosen from among the persons attending patients, and included both males and females with an age range of 35–50 years.

**Table 1:** Description of the study population by age, clinical and haematological parameters (Mean ± SD).

Characteristics	VL patients (n = 20)
Age (yrs)	32.31 ± 16.99
Sex	
Male	14
Female	6
Body weight (Kg)	45.90 ± 13.86
Spleen size (cm)	8.04 ± 2.76
Haemoglobin (g/dl)	7.95 ± 1.47
WBC count (cells/μl)	2840 ± 650
Albumin (gm/dl)	3.28 ± 0.71
Globulin (gm/dl)	4.897 ± 0.32
Albumin/Globulin	0.67
Platelet count (×10^3^)	142 ± 1.48
Parasite load (parasites/ml)	2549 ± 1213

### Ethics statement

The study was approved by and carried out under the guidelines of the Ethical Committee of the Indian Institute of Chemical Biology, Kolkata. All participants including ECs, NECs and patients or responsible adults provided written informed consent for the collection of samples and subsequent analysis.

### DNA isolation from blood and parasites

Blood samples were collected in heparinized tubes. DNA extraction was performed using QIAamp DNA Blood mini kits (Qiagen, Germany) from whole blood according to the manufacturer’s instructions. DNA was isolated from 400 μl of sample and eluted in 25 μl elution buffer. A stock solution of *L*. *donovani* DNA was also obtained by extraction (QIAamp DNA mini kit, Qiagen, Germany) from 2.5×10^6^ promastigotes. After eluting in 25 μl elution buffer, assuming the extraction was nearly 100% efficient, the DNA concentration corresponded to10^5^ parasites/μl. All the DNAs were stored at -20°C until use.

### Real-time PCR assay

SYBR Green-based real-time PCR was applied for quantification of the *Leishmania* parasites in patient blood. For accurate sensitivity, *L*. *donovani*-specific kinetoplast DNA was chosen as the target region. The PCR was performed in a final volume of 20 μl containing 10 μl of SYBR Green master mixture (2×) [Applied Biosystems (ABI), Carlsbad, CA, USA], 3 μl of MiliQ water, 5 μl of DNA template and 1 μl (50 pmol/μl) of forward and reverse primers, 5′-CTTTTCTGGTCCTCCGGGTAGG-3′ and 5′-CCACCCGGCCCTATTTTACACCAA-3′ (Integrated DNA Technologies, Coralville, IA, USA) respectively. To construct the standard curve ten-fold serially diluted *L*. *donovani* DNA stocks corresponding to 10^5^, 10^4^, 10^3^, 10^2^, 10, 1 parasite/μl were included in the assay. Amplification was conducted using a StepOnePlus Real-Time PCR system (ABI). The thermal cycling conditions included an initial incubation at 50°C for 2 min, followed by a 10 min denaturation at 95°C and 40 cycles at 95°C for 15 s and 60°C for 1 min each. Each sample was tested in duplicate. The calculation of the melting temperature of each amplicon was done directly by the software provided (StepOne Software). Negative controls with no template were included in each plate to deal with contamination issues. For all the real-time PCR reactions we used specific primers for the constitutively expressed beta actin gene (forward: 5’-GGCCAACCGCGAGAAGAT-3’; reverse: 5’-CGTCACCGGAGTCCATCAC-3’) as a quality control to verify the integrity of the DNA samples. Threshold cycle values (Ct) were calculated for patient samples by determining the point at which the fluorescence exceeded the threshold limit plotted against known concentrations of parasite DNA, and the parasite load of patient samples was then determined using the standard curve [3, 39].

### Preparation of *Leishmania* membrane antigens

*L*. *donovani* promastigote membrane antigens (LAg) were prepared as previously described [40] and stored at –70°C. The amount of protein thus obtained was determined by the method of Lowry *et al*. [41]. The endotoxin level in LAg was measured using the chromogenic Limulus Amebocyte Lysate (LAL) assay kit (QCL-1000; Lonza, Walkersville, MD, USA) according to the manufacturer's recommendations and was found to be virtually endotoxin free (<10 endotoxin units per mg of protein).

### Cytokine analysis

Plasma and PBMCs were isolated from the blood samples as described previously [17]. Plasma was then stored at -20°C until use and PBMCs were cultured with LAg (12.5 μg/ml). After 96 hrs, the supernatants of the cultures were collected and stored at -70°C until use. IFNγ, IL-12, IL-10 (BD OptEIA ELISA kit; BD Biosciences, San Jose, CA, USA), TGFβ, IL-17 (eBioscience, Inc., San Diego, CA, USA) and IL-22 (R&D Systems, ELISA DuoSet, McKinley Place, MN, USA) were measured in plasma samples according to manufacturers’ instructions. IFNγ, IL-12, IL-10 and TGFβ were also measured in culture supernatants following the same protocol. The color reaction was performed using avidin-HRP and tetramethylbenzidine, and read at OD_450_ [17, 20].

### Flow cytometry: Intracellular staining and absolute counting

For intracellular detection of cytokines, samples having a minimum cell count of 4 × 10^6^ were chosen. PBMCs from such samples were stimulated with 5 μl of 10 ng/μl phorbol 12-myristate 13-acetate (PMA) and 1 μl of 1 mg/ml ionomycin (Sigma, Saint Louis, MO, USA) at 37°C and under 5% CO_2_ for 2 hrs. Brefeldin A (10 mg/ml; Sigma) was added, and the samples were incubated for an additional 1 hr. Initially, stimulating abilities were compared between PMA/ionomycin, LAg and a combination of both PMA/ionomycin and LAg with all the alternatives triggering cytokines to similar levels (S1 Fig). Our observations were consistent with those of Owens *et al*. [42], who also failed to differentiate between antigenic and mitogenic stimulation when used with spleen cells from an experimental VL model. PMA/ionomycin was chosen as a standard triggering agent for its shortest stimulation time. The exact interval for the addition of Brefeldin A was standardized through a time kinetics study, in which negligible amounts of cytokines were found to be present in the culture supernatant 2 hr after the addition of PMA/ionomycin. The cells were stained with CD4-phycoerythrin-cyanine 7 conjugate (PE-Cy7, BD Biosciences) and CD25-allophycocyanin-cyanine 7 conjugate (APC-Cy7, BD Biosciences) to identify the CD4^+^CD25^+^ T cell population. In our conditions APC-Cy7 conjugated CD25 was able to produce a strong signal and a distinct demarcation between CD25^l^°^w^ and CD25^hi^ populations. After washing with FACS buffer [0.2 M PBS (17.8 g Na_2_HPO_4_, 6.9 g NaH_2_PO_4_, 9% NaCl, and distilled water to obtain 1 L, pH 7.2) and 1% FBS (Gibco, Life Technologies, Grand Island, NY, USA)], the samples were fixed and permeabilized by adding 100 μl of Cytofix/Cytoperm solution (BD Biosciences) in the dark for 20 min. After centrifugation, the supernatant was discarded, and the cells were intracellularly stained to detect FoxP3-fluorescein isothiocyanate (FITC, BD Biosciences), IL-10- allophycocyanin (APC, BD Biosciences), TGFβ-phycoerythrin (PE, BD Biosciences) and IFNγ-PE-Cy7 (BD Biosciences) for 30 mins in the dark. The samples were washed with FACS buffer supplemented with 0.1% Saponin (Sigma). Cell acquisition and analysis were performed using FACS Diva Software (BD) on a FACSCanto flow cytometer (BD). Prior to the addition of Cytofix/Cytoperm a small aliquot of the sample was analyzed for viability through PI staining and cells were found to be almost 95% viable. To further exclude any dead cells or debris we performed doublet discrimination through forward scatter-area/ forward scatter-width gating along with normal forward scatter-area/ side scatter-area gating. T cells were identified based on CD4-PE-Cy7 staining and different forward scatter and side scatter parameters. In total, 50,000 events were acquired. Regulatory T cells were gated as FoxP3-positive cells among CD4^+^CD25^+^ population, and the percentages of cells producing IL-10 or TGFβ were determined using thresholds based on unstimulated samples (S2 Fig). Isotype-matched controls (BD Biosciences) for FITC, PE, APC and PE-Cy7 were used in all staining experiments [42, 43]. Negative controls (cells plus all reagents minus PMA/ionomycin) were also used in the intracellular experiments as a background control to rule out the effect of any nonspecific stimulation [44].

For absolute counting, 50 μl of heparin-treated whole blood samples were taken in TruCount tubes (BD Biosciences) and incubated for 30 mins in the dark at room temperature with 5 μl each of CD45-PE-Cy7, CD3-peridinin chlorophyll (PerCP), CD4-APC, CD8-FITC and CD25-PE (BD Biosciences). Erythrocytes were lysed with 450 μl lysing solution (BD Biosciences) according to the manufacturer’s protocol. Cell acquisition and analysis were performed using FACS Diva Software (BD) on a FACSCanto flow cytometer (BD). For TruCount tubes, the analysis software calculated the percentage of lymphocytes that were positive for the subset of interest (CD3, CD4, CD8, CD4^+^CD25^+^ and CD4^+^CD25^high^) by identifying lymphocytes as being positive for CD45 but with low side scatter (S3 Fig) [45]. Absolute numbers were calculated as (number of cells of interest counted/number of beads counted) × (total number of beads in tube [from manufacturer]/50 μl [volume of blood tested]) [46].

### Statistical analysis

Correlation was evaluated using Spearman/Pearson correlation test. All data are presented as mean ± SD and a difference in mean values was considered significant when the *P* value was <0.05. Statistical analysis was carried out using GraphPad Prism 5 (GraphPad Software, Inc., San Diego, CA, USA).

## Results

### Real-time PCR assay for parasite quantification

**Fig 1:**
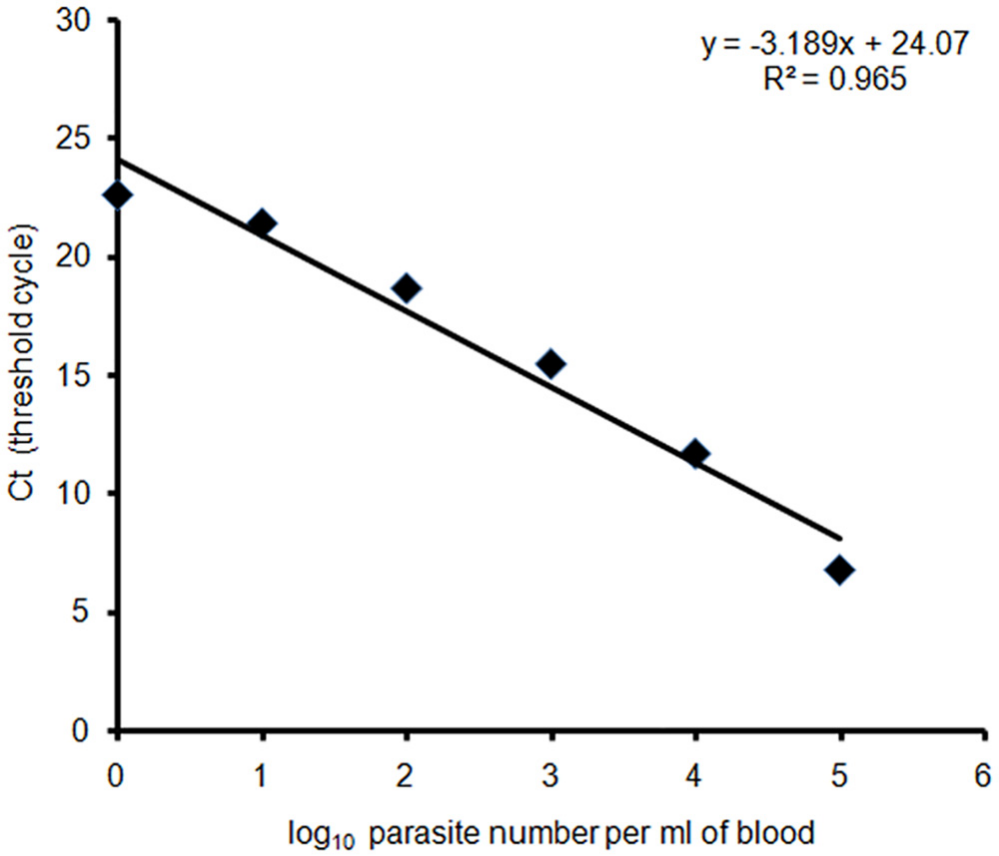
Standard curve for quantification of *Leishmania* parasites by real-time PCR. The standard curve was obtained by amplification of *Leishmania* DNA from 10^5^ to 1 parasites. Linear regression analysis rendered the equation y = -3.189x+24.07 (R^2^ = 0.965). Correlation of disease manifestations with parasite load.

The real-time PCR assay was initially tested for its specificity of detecting *L*. *donovani* parasites. A sequential dilution of *L*. *donovani* culture with corresponding final concentrations ranging from 10^5^ to 1 parasites was carried out and DNA was extracted from each dilution. Ct values obtained by subjecting those DNA samples to real-time PCR were used to construct a standard curve for determining parasite load in patient blood. The standard curve, containing 6 logarithmic parasitic DNA dilutions, was linear with a correlation coefficient (r^2^) of 0.965 (Fig 1). The Ct values, as obtained using real-time PCR assay, were 1.91, 2.46, 2.84, 2.93, 3.06 and 3.12 for the six different concentrations. Melting temperature (T_m_) of the samples, as calculated through melt curve analysis, was found to be 84.32 ± 0.09°C (mean T_m_ values ± standard error) and was comparable between all the samples (S4 Fig). Percentage PCR efficiency was found to be 105.86%, using the following equation: %Efficiency = [10^(-1/sl^°^pe)^-1] × 100% and slope of the standard curve (-3.189). Negative control (water instead of template DNA) was included in each PCR assay to control for any contamination issues.

**Fig 2:**
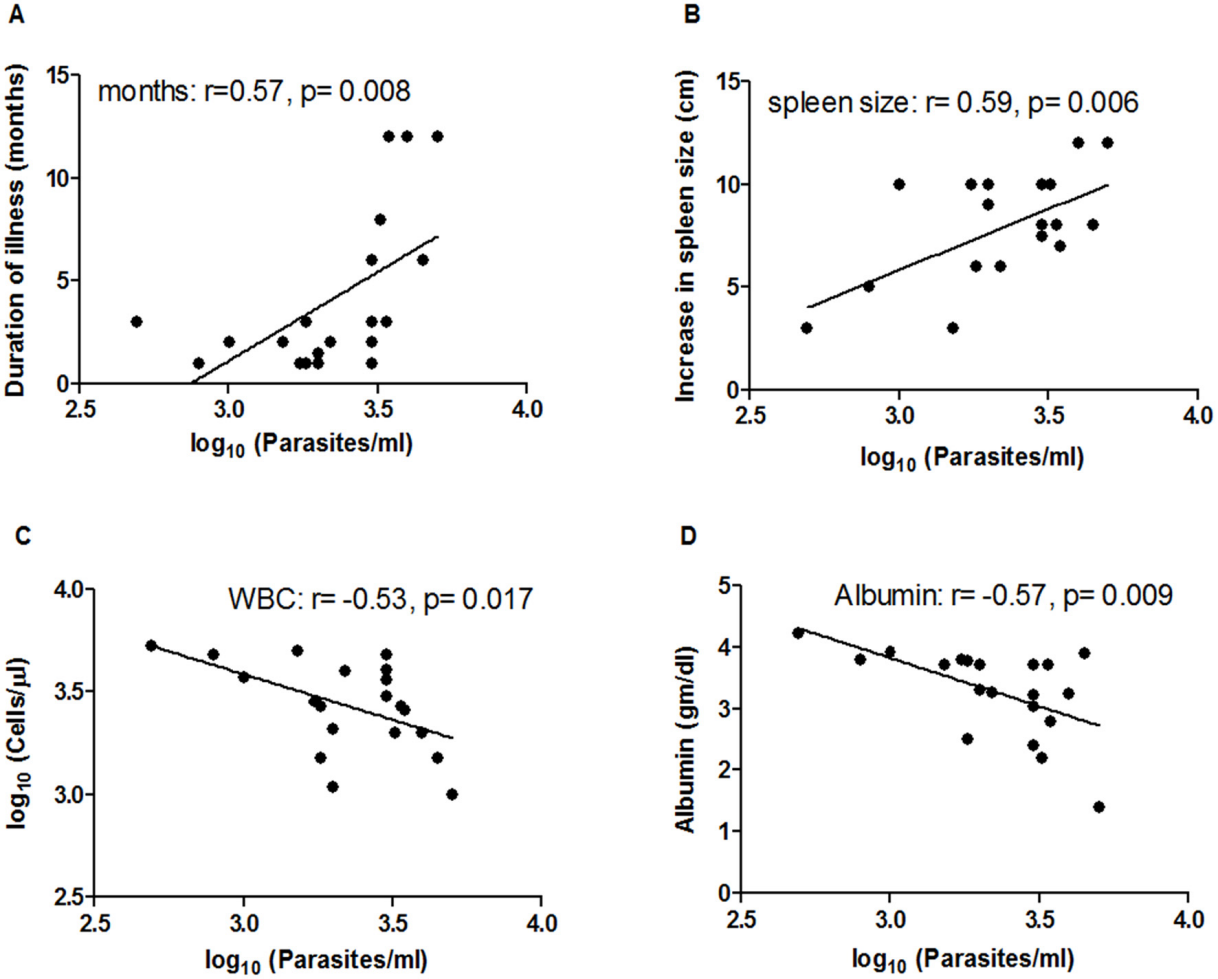
Correlation of parasite load with different clinical and hematological parameters of VL patients (n = 20). Duration of illness (months, panel A), increase in spleen size (cm, panel B), white blood cell counts (cells/μl, panel C) and albumin levels (gm/dl, panel D) of VL patients were obtained from the hospital. Parasite loads (Parasites/ml) were determined by real-time PCR. Correlation was calculated using the Spearman/Pearson correlation test. Diagonal lines represent linear regression.

Determination of parasite loads in blood samples was performed in all the VL patients (n = 20). The average parasite load in circulation was 2549 parasites/ml, ranging from 487.5 parasites/ml to 5000 parasites/ml (Table 2). No parasites were detected in blood samples of the healthy controls (both NECs and ECs). To evaluate the association of parasite load with disease pathology different clinical as well as hematological parameters were considered (Table 2). Significant positive correlations were found between parasite load and duration of illness [length of time the patients suffered from fever before being admitted to hospital] (r = 0.57, p = 0.008) (Fig 2A) as well as spleen size (r = 0.59, p = 0.006) (Fig 2B), whereas the correlation was significantly negative with white blood cell counts (r = -0.53, p = 0.017) (Fig 2C) and albumin levels (r = -0.57, p = 0.009) (Fig 2D).

**Table 2:** Results of *Leishmania* DNA quantification in blood samples and clinical parameters of VL patients.

Patient nos.	Parasite load/ ml of blood	Duration of illness (mo)	Spleen size (cm)	WBC (cells/μl)	Albumin (gm/dl)
**1.**	1820	3.0	6.0	1500	3.78
**2.**	1995	1.5	10.0	1100	3.30
**3.**	1514	2.0	3.0	5000	3.70
**4.**	1738	1.0	10.0	2800	3.80
**5.**	3020	3.0	10.0	4800	3.22
**6.**	3020	1.0	7.5	4100	3.70
**7.**	3020	6.0	7.5	3700	3.04
**8.**	4467	6.0	8.0	1500	3.90
**9.**	794	1.0	5.0	4800	3.80
**10.**	2188	2.0	6.0	4000	3.25
**11.**	487.5	3.0	3.0	5200	4.22
**12.**	1000	2.0	10.0	3700	3.92
**13.**	3981	12.0	12.0	2000	3.24
**14.**	5000	12.0	12.0	1000	1.40
**15.**	3388	3.0	8.0	2700	3.70
**16.**	3467	12.0	7.0	2600	2.80
**17.**	3020	2.0	8.0	3020	2.40
**18.**	1820	1.0	6.0	2700	2.50
**19.**	1995	1.0	9.0	2100	3.70
**20.**	3236	8.0	10.0	2000	2.20

### Correlation of immune response with parasite load

**Fig 3:**
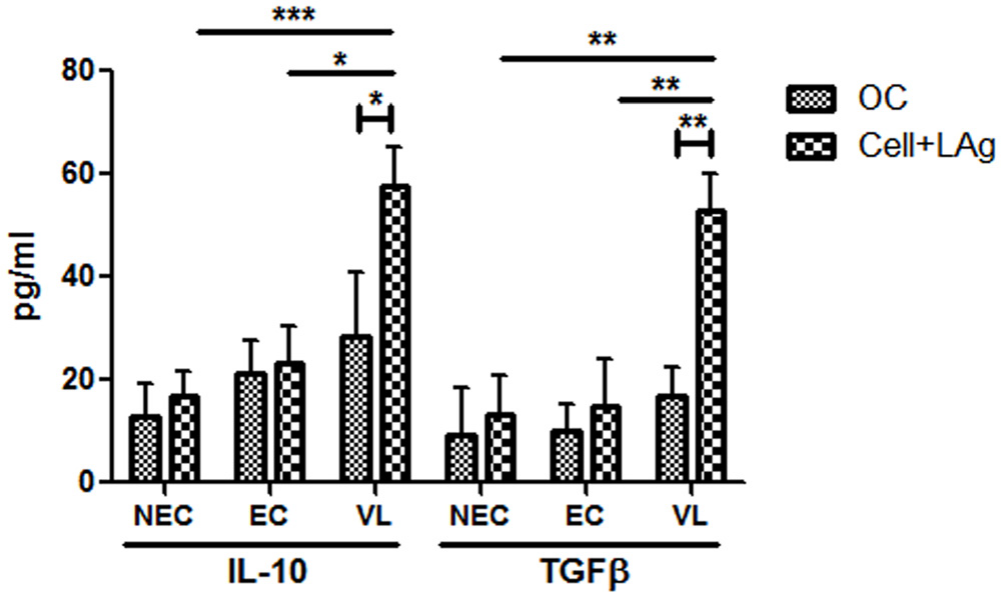
*Leishmania*-specific cell-mediated immune response in VL patients and healthy controls (ECs and NECs). PBMCs were isolated from VL patients (n = 10), ECs (n = 5) and NECs (n = 10). Cells (2×10^5^/well) were stimulated with 12.5 μg/ml of LAg for 4 days and cytokines from supernatants were measured by ELISA. IL-10 and TGFβ levels (pg/ml) were compared between LAg-stimulated and unstimulated samples (only cell, OC) from VL patients as well as LAg-stimulated samples from ECs and NECs. Data are represented as mean ± SE. *P* values were calculated using non-parametric Mann-Whitney *U* test and Unpaired t test; *P*<0.05 was considered significant.

**Fig 4:**
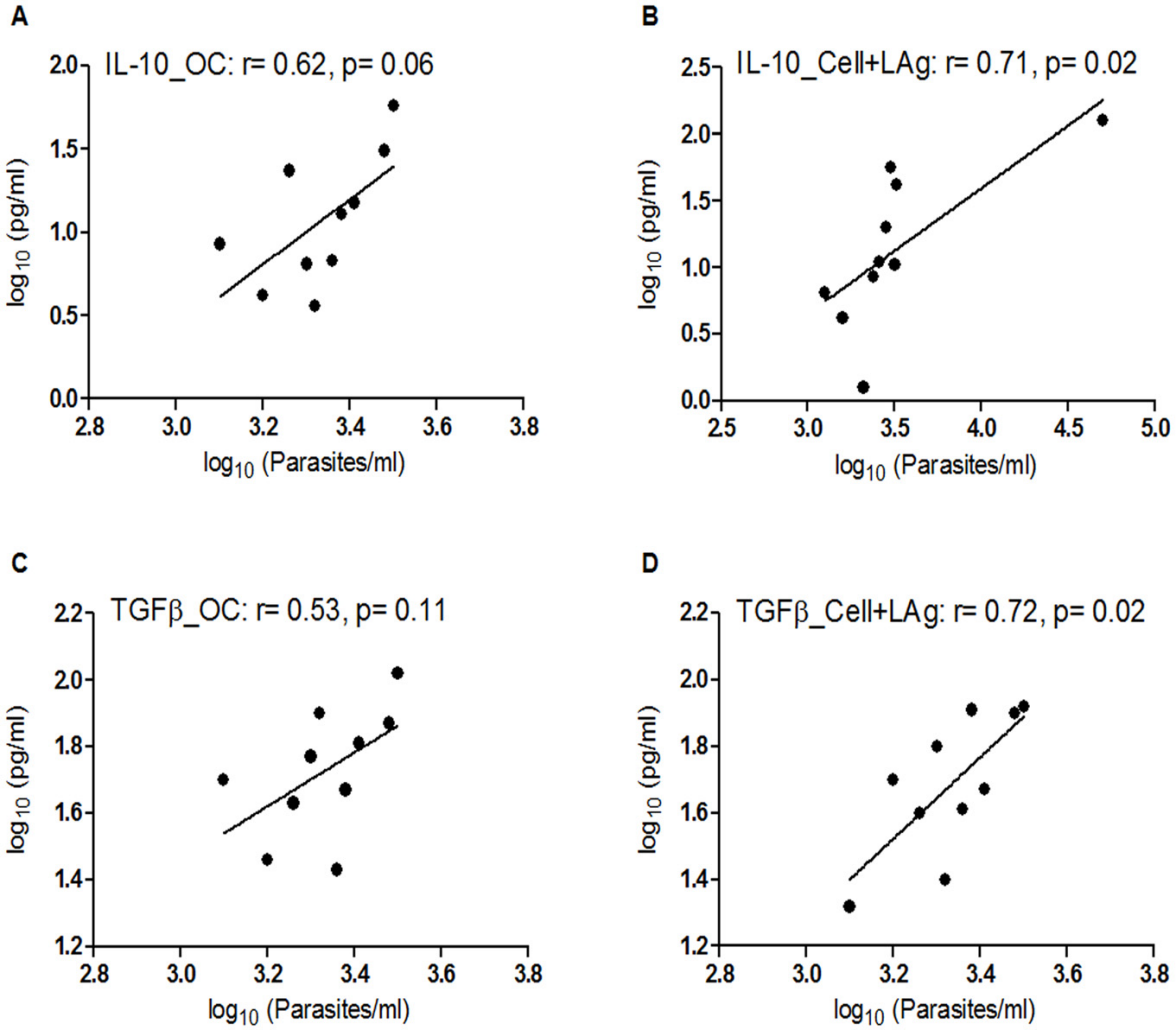
Correlation of different cytokines in culture supernatants of VL patients (n = 10) with parasite load. The unstimulated and LAg-stimulated levels (pg/ml) of IL-10 (panels A and B) and TGFβ (panels C and D) in PBMCs of VL patients were measured by ELISA, and parasite loads (Parasites/ml) were determined by real-time PCR. Correlation was calculated using Spearman/Pearson correlation test. Diagonal lines represent linear regression.

Expression levels of IFNγ, IL-12, IL-10, TGFβ, IL-17 and IL-22 were analyzed in plasma of VL patients (n = 20) by ELISA (S1 Table). Patients harboring higher number of parasites seemed to produce higher levels of plasma cytokines IL-10, TGFβ and IL-17. Indeed, their circulating levels were found to correlate strongly and significantly with parasite load (r = 0.52; p = 0.02 for IL-10, r = 0.53; p = 0.018 for TGFβ and r = 0.51; p = 0.02 for IL-17). The correlation, however, was not so strong for the other three cytokines tested (r = 0.23 for IFNγ, 0.25 for IL-12 and 0.25 for IL-22). However, since VL is immunosuppressive and often involves co-infection, circulating cytokine levels do not always accurately represent the actual disease scenario. Thus investigation of the correlation between parasite load and antigen specific levels of cytokines in PBMCs of VL patients that were already found to be elevated in plasma was carried out. PBMCs were collected from VL patients (n = 10) having a minimum cell count of 4 × 10^6^ and cultured in vitro in the presence of LAg for 4 days as reported earlier [20, 47, 48]. LAg-stimulated culture supernatants contained significantly higher levels of IL-10 (mean level of 57.8 pg/ml) and TGFβ (mean level of 53 pg/ml) in comparison to unstimulated cultures (mean levels of 28.5 pg/ml for IL-10 and 17 pg/ml for TGFβ) (Fig 3). Moreover, when compared with ECs (n = 5) and NECs (n = 10), VL patients were found to produce significantly higher levels of both LAg stimulated IL-10 (p = 0.013 for EC and 0.0002 for NEC) and TGFβ (p = 0.0075 for EC and 0.0012 for NEC), suggesting the specificity of LAg towards infected individuals (Fig 3). Levels of IL-10 and TGFβ produced by unstimulated cells correlated positively (r = 0.62 for IL-10 and r = 0.53 for TGFβ) with parasite load (Fig 4A and 4C), although the correlation was not statistically significant. However, the correlation was significant when IL-10 (r = 0.71, p = 0.02) and TGFβ (r = 0.72, p = 0.02) produced by LAg stimulated PBMCs were plotted against parasite load (Fig 4B and 4D). Correlation between parasite load and unstimulated levels of IFNγ and IL-12 could not be performed due to the low titres of these cytokines and while the association between LAg stimulated levels of both these cytokines with parasite load was not so strong (r = -0.4 for IFNγ and -0.06 for IL-12), a negative slope in either cases signified their inhibitory role in parasite multiplication (S5A and S5B Fig). Correlation of parasite load with antigen specific IL-17 and IL-22, however, also could not be performed due to the low titre of these cytokines in both unstimulated and LAg stimulated PBMCs of VL patients.

**Fig 5:**
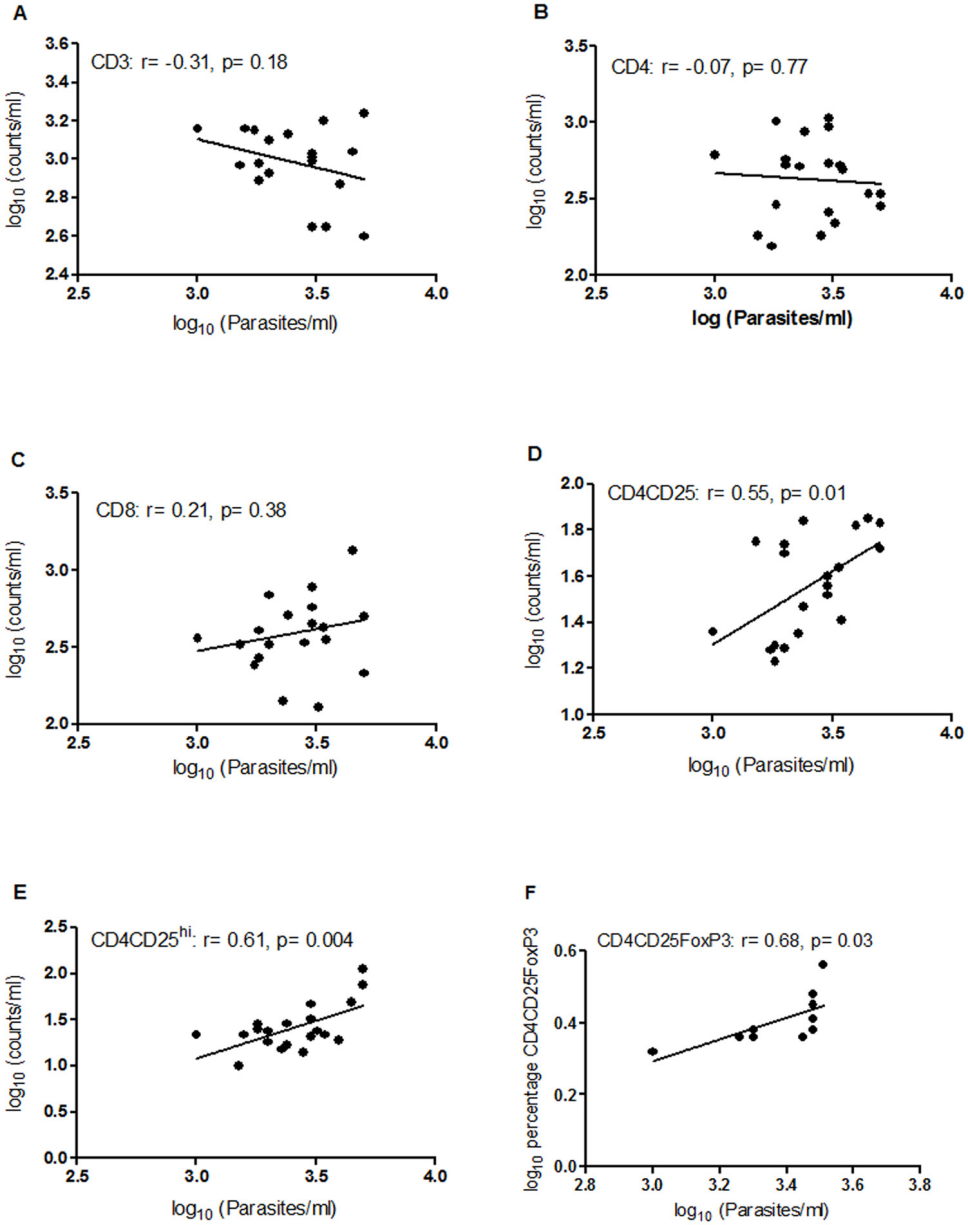
Correlation of different T cell subtypes and CD4CD25FoxP3 with parasite load in VL patients. Absolute numbers of CD3 (panel A), CD4 (panel B), CD8 (panel C), CD4CD25 (panel D), and CD4CD25^hi^ (panel E) were determined by flow cytometry in whole blood samples of VL patients (n = 20). Percentages of CD4CD25FoxP3-positive cells (panel F) were calculated by flow cytometry in PBMCs of VL patients (n = 10). Parasite loads (Parasites/ml) were determined by real-time PCR. Correlation was calculated using Spearman/Pearson correlation test. Diagonal lines represent linear regression.

The increase in absolute number of circulating CD4^+^CD25^+^ Treg cells observed in VL patients suggested its probable role during *L*. *donovani* infection. To further elucidate if there is a direct association between these two variables, the absolute numbers of CD4^+^CD25^+^ Treg cells were compared with parasite load (Fig 5D). The data clearly show that the number of CD4^+^CD25^+^ Treg cells is positively correlated with parasite load (r = 0.55, p = 0.01) in VL patients. Additionally, since high expression of CD25 has been reported as a more specific marker for Tregs [49], we also assessed CD4^+^CD25^hi^ Treg cells and found their numbers to be significantly correlated (r = 0.61, p = 0.004) with increasing levels of parasite load (Fig 5E). The best characterized Treg cell is, however, defined by the expression of its transcriptional regulator, FoxP3 [11]. To evaluate the role of this subpopulation in active VL we investigated the association between frequency of CD4^+^CD25^+^FoxP3^+^ Treg cells with parasite load in patients (n = 10) having at least 4 × 10^6^ cells. Our data exhibited a significant positive correlation (r = 0.68, p = 0.03) between parasite load and percentage of CD4^+^CD25^+^FoxP3^+^ T cells (Fig 5F). These observations confirm the involvement of CD4^+^CD25^+^FoxP3^+^ Treg cells in VL pathogenesis. Although no significant correlation with parasite load was observed for overall CD3 (Fig 5A), CD4 (Fig 5B) and CD8 (Fig 5C) T cell subsets, the regression line showed a negative slope for CD3 (Fig 5A) and CD4 (Fig 5B) implying a decrease in their numbers with increasing parasite load whereas a positive slope for CD8 (Fig 5C) indicates an increase in CD8 count with increasing number of parasites.

**Fig 6:**
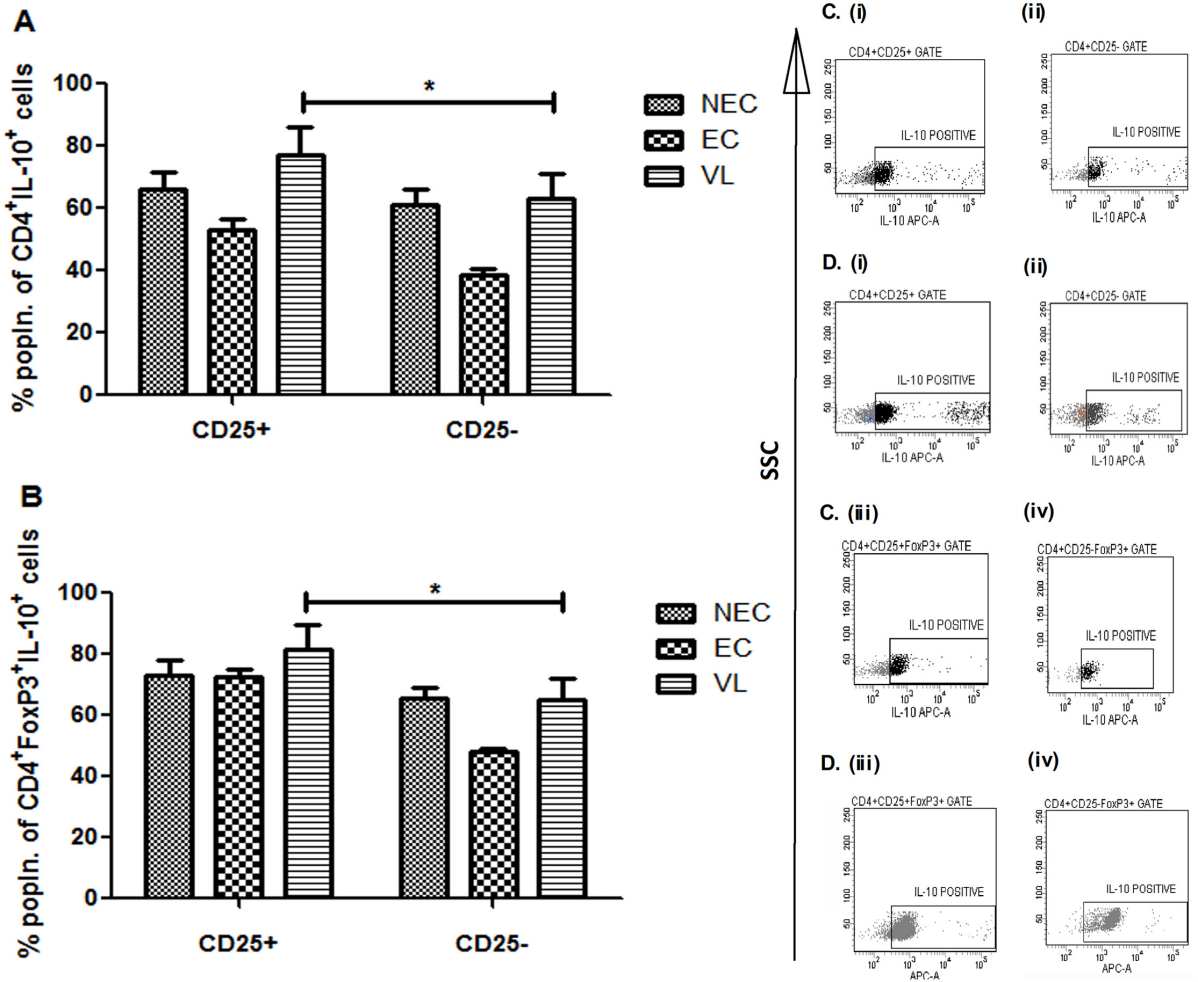
Identification of cellular sources of IL-10 in PBMCs of VL patients (n = 10), ECs (n = 5) and NECs (n = 5). Total PBMCs were freshly cultured in the presence of PMA (50 ng/μl), ionomycin (1 μg/μl) for 2 hrs and for additional 1 hr in presence of brefeldin A (10 μg/μl) before staining. (A) Percentages of CD25+ and CD25− cells among CD4+IL-10+ cells. (B) Percentages of CD25+ and CD25− cells among CD4+FoxP3+IL-10+ cells. Data are represented as mean ± SE. *P* values were calculated using Wilcoxon matched pairs signed rank test for paired samples; *P*<0.05 was considered significant. (C), (i)-(iv) Data showing one representative healthy control. (D), (i)-(iv) Data showing one representative active VL patient.

**Fig 7:**
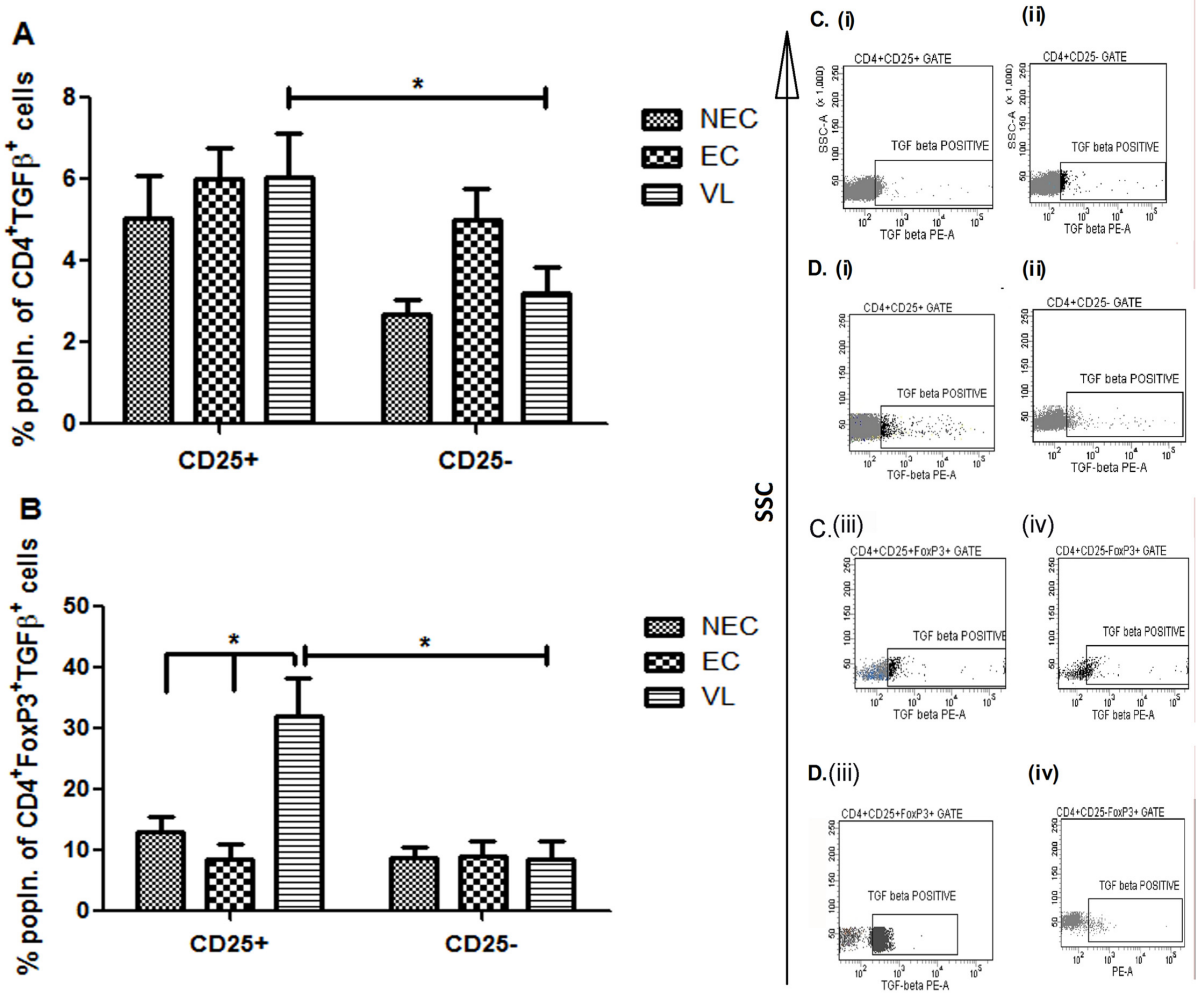
Identification of cellular sources of TGFβ in PBMCs of VL patients (n = 10), ECs (n = 5) and NECs (n = 5). Total PBMCs were freshly cultured in the presence of PMA (50 ng/μl), ionomycin (1 μg/μl) for 2 hrs and for additional 1 hr in presence of brefeldin A (10μg/μl) before staining. (A) Percentages of CD25+ and CD25− cells among CD4+TGFβ+ cells. (B) Percentages of CD25+ and CD25− cells among CD4+FoxP3+TGFβ+ cells. Data are represented as mean ± SE. *P* values were calculated using Wilcoxon matched pairs signed rank test for paired samples; *P*<0.05 was considered significant. (C), (i)-(iv) Data showing one representative healthy control. (D), (i)-(iv) Data showing one representative active VL patient.

Significant positive correlation between parasite load and different cytokines as well as cellular subsets provided us the opportunity to investigate the link between these immune components. Since the cellular origins of IL-10 and TGFβ during active VL remains obscure, we examined CD4^+^CD25^+^ and CD4^+^CD25^+^FoxP3^+^ Treg cells for their potential role as sources of these disease promoting cytokines. To understand the scenario, we first analyzed the expression of PMA/ionomycin–activated, IL-10–producing CD25 in CD4 and CD4^+^FoxP3^+^ cells from patients with active VL (n = 10) as well as from ECs (n = 5) and NECs (n = 5). Although the percentage of IL-10 produced by CD25-positive and negative populations was comparable between healthy controls (both ECs and NECs) (Fig 6A and 6B), both CD4^+^CD25^+^ and CD4^+^CD25^+^FoxP3^+^ cells of infected individuals were found to secrete significantly (p = 0.013 for CD4^+^CD25^+^ and p = 0.049 for CD4^+^CD25^+^FoxP3^+^) higher levels of IL-10 in comparison to the corresponding CD25-negative populations (Fig 6A and 6B). Fig 6C(i)–6C(iv) are representative of one healthy control and Fig 6D(i)–6D(iv) are representative of one active VL case, respectively. We then focused our study on probable source(s) of TGFβ in VL patients and there also found CD4^+^CD25^+^ and CD4^+^CD25^+^FoxP3^+^ cells to secrete significantly (p<0.05) higher levels of this cytokine in comparison to CD25-negative cells (Fig 7A and 7B). The percentage of TGFβ secreting CD4^+^CD25^+^FoxP3^+^ cells (32.06%) (Fig 7B), however, was significantly higher than that of CD4^+^CD25^+^ cells (6.04%) (Fig 7A). Moreover, the percentage of TGFβ producing CD4^+^CD25^+^FoxP3^+^ cells (32.06%) was also found to be significantly (p<0.05) higher than the similar population in ECs (12.9%) as well as NECs (13.08%) (Fig 7B) reaffirming our previous observations with circulating levels of TGFβ (Fig 3). Fig 7C(i)–7C(iv) are representative of one healthy control and Fig 7D(i)–7D(iv) are representative of one active VL case, respectively. Additionally, our observation with IFNγ yielded similar results, as both CD4^+^CD25^+^ and CD4^+^CD25^+^FoxP3^+^ cells were found to secrete significantly higher (p<0.01) levels of this cytokine in comparison to corresponding CD25^-^ cells (S6A and S6B Fig). To the best of our knowledge this is the first report regarding the involvement of CD4^+^CD25^+^FoxP3^+^ Tregs as a cellular source of TGFβ in VL patients. Taken together, our results showed that among PBMCs from *L*. *donovani* infected patients with VL, CD4^+^CD25^+^, or more specifically CD4^+^CD25^+^FoxP3^+^ Treg cells, are the most important sources of the disease promoting cytokines IL-10 and TGFβ.

## Discussion

The present study investigated the correlation between infective parasites and different immune components associated with VL and assessed the impact of their interplay in predicting the disease outcome. Interestingly, our results revealed a strong correlation of increasing parasite load with different clinical parameters implying disease manifestations to be linked with the number of infective parasites as well as symptoms like splenomegaly, leukocytopenia and hypoalbuminemia. Our data also demonstrated a positive association between parasite load and elevated levels of IL-10 and TGFβ along with absolute numbers of CD4^+^CD25^+^ T cells and percentages of CD4^+^CD25^+^FoxP3^+^ positive T cells suggesting the involvement of these cytokines and Treg cells in course of disease. Taking a cue from this parasitological evidence, we went further and were able to identify the significance of CD4^+^CD25^+^FoxP3^+^ Treg cells in secretion of these cytokines, in comparison to corresponding CD25^-^ T cells.

The course of VL depends on the interplay between the host and the infecting pathogen in which the survival of parasites is primarily regulated by the type of cytokine being secreted upon and their interaction with host immune cells. Therefore, we analyzed some important disease promoting/protective as well as Th17 cytokines in VL patients to understand the influence of the immune response on parasite load. IL-10 has been well known for its role in VL pathogenesis and a significant correlation was already reported between parasite load and plasma IL-10 levels in VL patients [3]. Our study with plasma IL-10 also supported this notion, and an extreme case having the highest level of IL-10 was presented with very high parasite load (4500 parasites/ml of blood with 350 pg/ml plasma IL-10). However, since VL patients are often co-infected with other pathogens such as mycobacteria [50], which could also result in elevated plasma lipopolysaccharide concentration and subsequent impairment in immune effector function [51], or a systemic inflammatory response syndrome called sepsis [52], total plasma cytokine profiles might not be a conclusive representation of infection. Therefore, we investigated the recall response in LAg stimulated PBMCs of some of these patients and found even a stronger correlation between parasite load and IL-10 levels in these patients. To the best of our knowledge, this is the first report of the correlation between number of parasites and antigen-specific production of IL-10 in the human VL scenario. Another cytokine which could contribute to the pathology of human VL progression is TGFβ [20]. It has been shown to impart down-regulatory effects on macrophages and its blockade could restrict parasite progression in these cells [53]. Since there has been no previous report on its association with parasite load in human VL, we examined the correlation between parasite load and circulating/antigen specific TGFβ levels in VL patients. The correlation was significant in plasma and appeared stronger in LAg stimulated culture supernatants of VL patients, confirming the role of TGFβ in parasite multiplication and evolution of disease. A correlation, although not statistically significant, was also recorded between parasite load and both these cytokines (IL-10 and TGFβ) produced by unstimulated cells, indicating that *in vitro* production of these cytokines partly resulted from stimuli received *in vivo*. IL-10 has also been reported to synergize with TGFβ to inhibit macrophage microbicidial activity thereby enhancing parasite growth and survival [54]. Our data supports this hypothesis as production of both IL-10 and TGFβ were clearly correlated with active disease. The role of Th17 cells and their cytokines in leishmaniasis remain obscure as both protective and pathogenic responses have been influenced by them [55]. In human VL, there are very few reports, and while in one case IL-17 levels were found to be higher in VL resistant individuals [36], in other instances it was found to be elevated in VL susceptible patients [56, 57]. We evaluated the association of parasite load with plasma IL-17 and found a positive correlation between them. However, as already explained, total circulating levels of any cytokine was not enough to depict the actual disease scenario. In fact, IL-17 level in plasma was found to be triggered in VL patients co-infected with malaria [57] and since disease resistance was associated with elevation in *L*. *donovani* antigen stimulated IL-17 levels [36], a more precise antigen-specific response would be desirable to draw any conclusion in this respect.

Although IL-10 and TGFβ have been reported to contribute to VL pathogenesis [58], information regarding the cellular subtypes associated with the elevation of these cytokines during infection remains limited. In human CL, it has been observed that T cells expressing IL-10 and/or TGFβ are mostly natural Treg cells and thus can be considered as the source of these disease promoting cytokines [28, 29]. However, in human VL the evidence is not conclusive. While some studies at flow cytometric as well as transcriptional levels failed to show any significant increase in CD4^+^CD25^+^FoxP3^+^ Treg cells during active VL [11, 59], other studies recorded increased percentages of CD4^+^CD25^+^ Treg cells in addition to upregulation in the levels of IL-10 and TGFβ in both VL and PKDL patients [20]. Furthermore mRNA levels of different Treg cell markers like CD25 and FoxP3 as well as IL-10 were also shown to increase in lesional tissues of PKDL patients, and this elevation exhibited a significant positive correlation with parasite load [60]. This evidence suggests CD4^+^CD25^+^FoxP3^+^ Treg cells to be one of the important sources of IL-10 in VL patients [32] and which may play significant role in favoring parasite survival. In the case of TGFβ, the cellular sources were reported to be mostly CD4^+^CD25^+^ T cells in *L*. *guyanensis* [31] and *L*. *major* [30] infected individuals. However, there is no direct evidence for the origin of TGFβ during active VL. Our experiments with absolute numbers of different T cell subsets revealed a significant positive correlation of parasite load with not only CD4^+^CD25^+^ T cells but also CD4^+^CD25^high^ T cells. Higher percentages of CD4^+^CD25^+^FoxP3^+^ T cells were also found to be significantly correlated with increased parasite load. Since the surface marker and transcriptional regulators for Treg cells were well expressed in VL patients and correlated significantly with parasite load, it could be logical to infer that the heightened Treg activity may be the key determinant for parasite survival and multiplication inside the host. In fact, the abundance of Treg cells, along with IL-10 and TGFβ, in VL patients observed in the present study, may be the main reason for large numbers of parasites inflicting disease aggravation. Although these data did not allow us to predict the sources of disease promoting cytokines during infection, it encouraged us to take a more direct approach to characterize Treg cells as an integral part of disease pathology. Indeed, our data on different CD4^+^ T subsets and their cytokine producing ability conclusively identified CD4^+^CD25^+^ or specifically CD4^+^CD25^+^FoxP3^+^ Treg cells to be an important source of IL-10, when compared to CD4^+^CD25^-^ or CD4^+^CD25^-^FoxP3^+^ populations, respectively, in PBMCs of active VL patients. We were also able, for the first time, to describe CD4^+^CD25^+^ cells or more precisely CD4^+^CD25^+^FoxP3^+^ Treg cells as one of the sources of circulating TGFβ in *L*. *donovani* infected VL patients.

Collectively our data suggest a possible role of Tregs, IL-10 and TGFβ in parasite establishment and course of disease progression among VL patients. The finding that CD4^+^CD25^+^FoxP3^+^ Treg cells could produce both IL-10 and TGFβ upon *L*. *donovani* infection is of prime importance in the knowledge of VL pathogenesis. Such findings could lead us to devise new vaccine strategies or immunotherapies against VL, which are important from the perception of eradication of parasites and cure of VL.

## Supporting Information

S1 Fig Comparison of IL-10 and TGFβ producing ability of different cellular subsets under different stimulations in active VL patients (n = 3). Total PBMCs were freshly cultured in the presence of (i) PMA (50 ng/μl), ionomycin (1 μg/μl) for 2 hrs and for additional 1 hr in presence of brefeldin A (10 μg/μl) [P/I], (ii) LAg (12.5 μg/ml) for 72 hrs. with brefeldin A for the last 1 hr. [LAg] and (iii) LAg (12.5 μg/ml) for 72 hrs. with PMA (50 ng/μl), ionomycin (1 μg/μl) for last 3 hrs. and brefeldin A for the last 1 hr [P/I+LAg]. Percentages of CD4+CD25-, CD4+CD25+, CD4+CD25-FoxP3+ and CD4+CD25+FoxP3+ cells producing (A) IL-10 and (B) TGFβ were calculated and compared with those of unstimulated cells. Data are represented as mean ± SE.
(TIF)

S2 Fig Strategy for the analysis of sources of IL-10 and TGFβ. Dot plots shown are representative of one VL patient. T cells were identified based on CD4-PE-Cy7 staining. For the analyses, regulatory T cells were gated as FoxP3-positive cells among CD4^+^CD25^+^ population, percentages of regulatory T cells producing IL-10 or TGFβ were determined with the quadrants established based on the unstained samples and isotype controls.
(TIF)

S3 Fig Strategy for the analysis of absolute T cell counts. Dot plots shown are representative of one VL patient. Lymphocytes were selected based on low SSC vs. high CD45-PE-Cy7 count and fifty thousand events were acquired. CD45+ gated lymphocytes were further analyzed for CD3, CD4, CD8, CD4CD25 and CD4CD25^hi^ expression. The cells with the phenotype CD4CD25 and CD4CD25^hi^ were considered to be Treg lymphocytes.
(TIF)

S4 Fig Melting curve analysis of selected samples. Data shown are for four sets of patient samples. Peaks of curves indicate the melting temperature of the amplicon.
(TIF)

S5 Fig Correlation of different cytokines in culture supernatants of VL patients (n = 10) with parasite load. The LAg-stimulated levels (pg/ml) of (A) IFNγ and (B) IL-12 in PBMCs of VL patients were measured by ELISA, and parasite loads (Parasites/ml) were determined by real-time PCR. Correlation was calculated using Spearman/Pearson correlation test. Diagonal lines represent linear regression.
(TIF)

S6 Fig Identification of cellular sources of IFNγ in PBMCs of VL patients (n = 10), ECs (n = 5) and NECs (n = 5). Total PBMCs were freshly cultured in the presence of PMA (50 ng/μl), ionomycin (1 μg/μl) for 2 hrs and for additional 1 hr in presence of brefeldin A (10 μg/μl) before staining. (A) Percentages of CD25+ and CD25− cells among CD4+ IFNγ+ cells. (B) Percentages of CD25+ and CD25− cells among CD4+FoxP3+ IFNγ+ cells. Data are represented as mean ± SE. *P* values were calculated using Wilcoxon matched pairs signed rank test for paired samples; *P*<0.05 was considered significant.
(TIF)

S1 Table Numerical representation of parasite load and different plasma cytokine profiles of VL patients (n = 20). (DOC)

S1 Checklist STROBE Checklist. (DOC)
