# Supplementary figures and images for: Induction of IL-10 and TGFβ from CD4+CD25+FoxP3+ T Cells Correlates with Parasite Load in Indian Kala-azar Patients Infected with Leishmania donovani
Source: PLoS Negl Trop Dis. 2016 Feb 1;10(2):e0004422. doi: 10.1371/journal.pntd.0004422 (PMC4735109; doi:10.1371/journal.pntd.0004422)

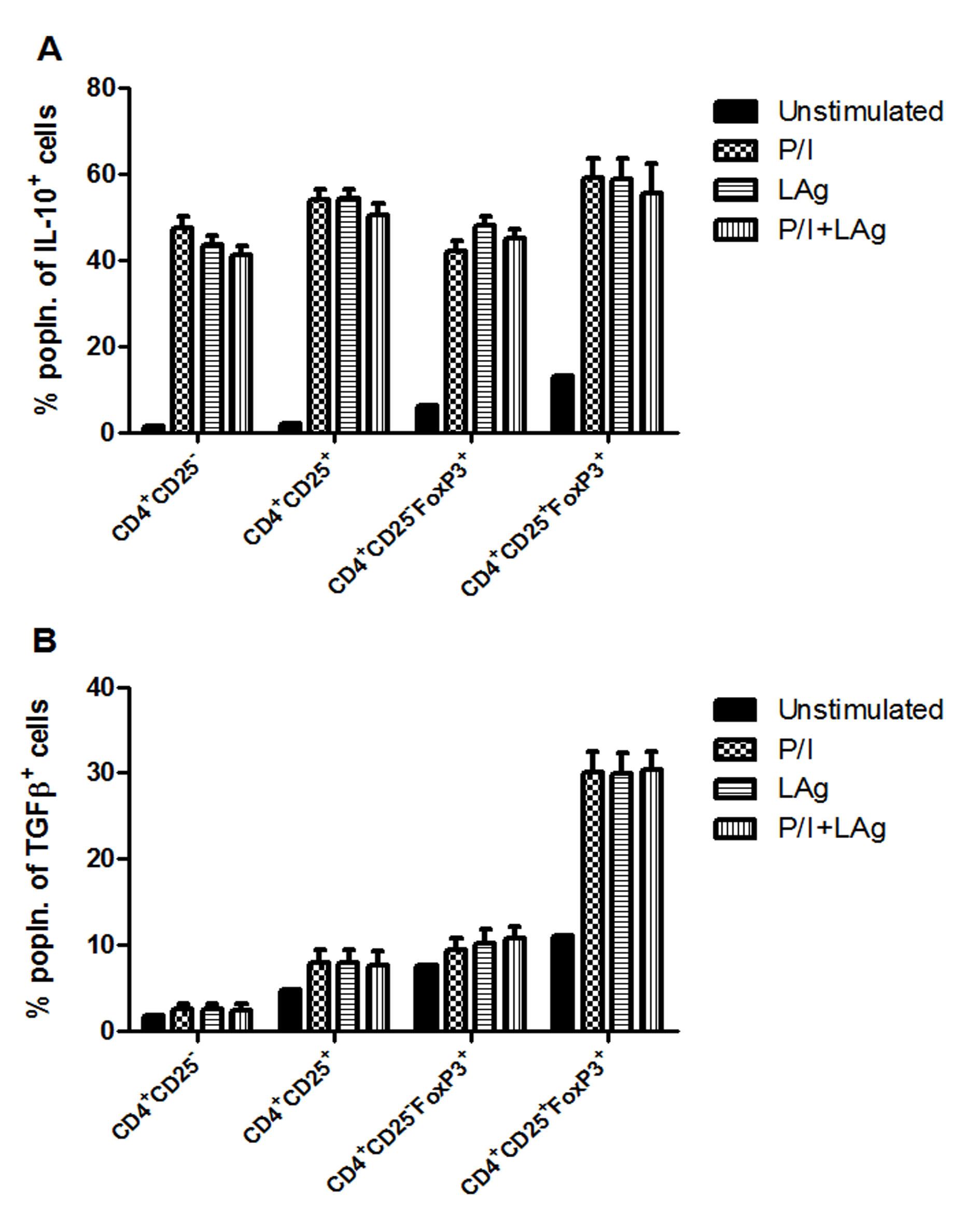

Supplement: S1 Fig — Total PBMCs were freshly cultured in the presence of (i) PMA (50 ng/μl), ionomycin (1 μg/μl) for 2 hrs and for additional 1 hr in presence of brefeldin A (10 μg/μl) [P/I], (ii) LAg (12.5 μg/ml) for 72 hrs. with brefeldin A for the last 1 hr. [LAg] and (iii) LAg (12.5 μg/ml) for 72 hrs. with PMA (50 ng/μl), ionomycin (1 μg/μl) for last 3 hrs. and brefeldin A for the last 1 hr [P/I+LAg]. Percentages of CD4+CD25-, CD4+CD25+, CD4+CD25-FoxP3+ and CD4+CD25+FoxP3+ cells producing (A) IL-10 and (B) TGFβ were calculated and compared with those of unstimulated cells. Data are represented as mean ± SE. (TIF) [file pntd.0004422.s001.tif]

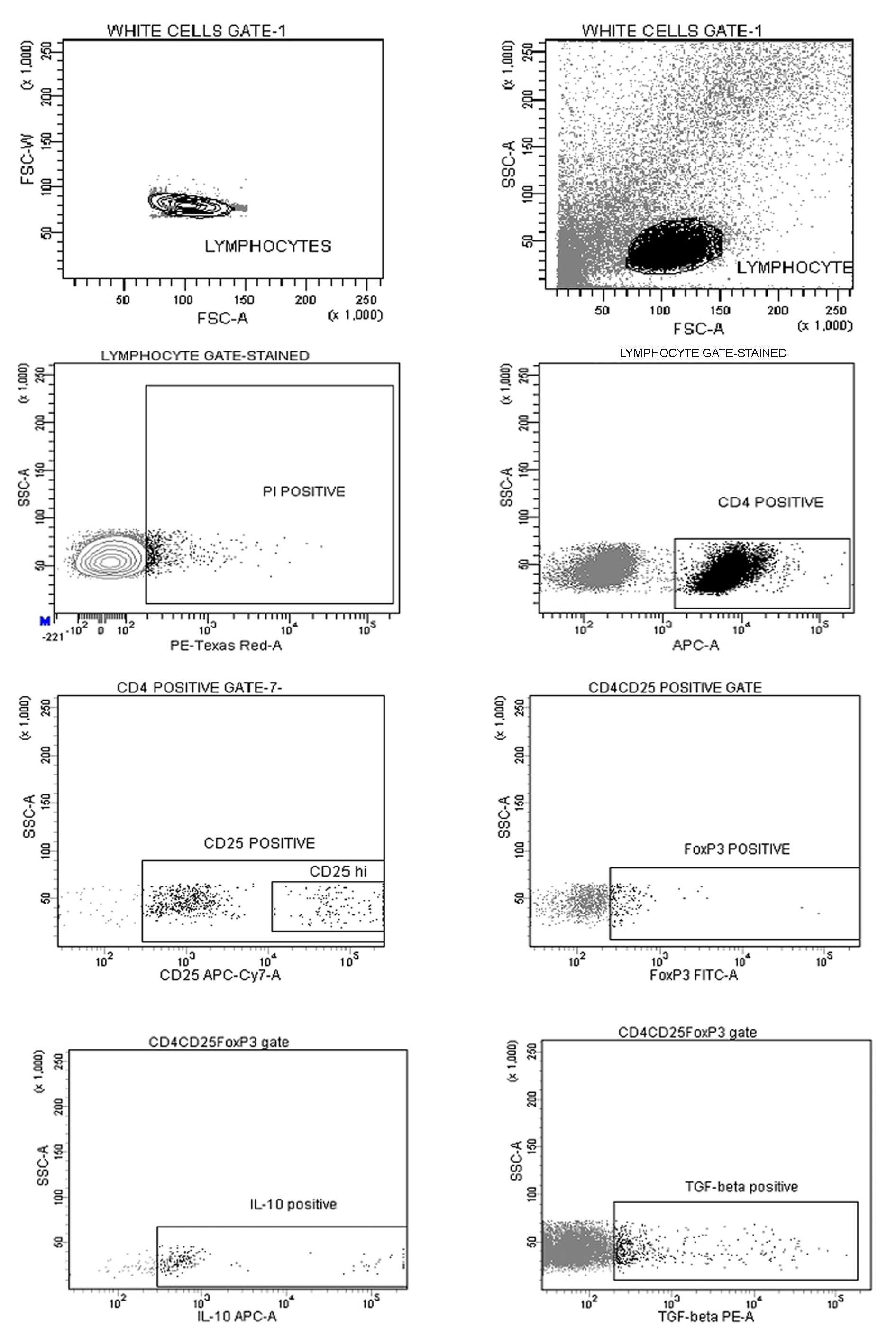

Supplement: S2 Fig — Dot plots shown are representative of one VL patient. T cells were identified based on CD4-PE-Cy7 staining. For the analyses, regulatory T cells were gated as FoxP3-positive cells among CD4+CD25+ population, percentages of regulatory T cells producing IL-10 or TGFβ were determined with the quadrants established based on the unstained samples and isotype controls. (TIF) [file pntd.0004422.s002.tif]

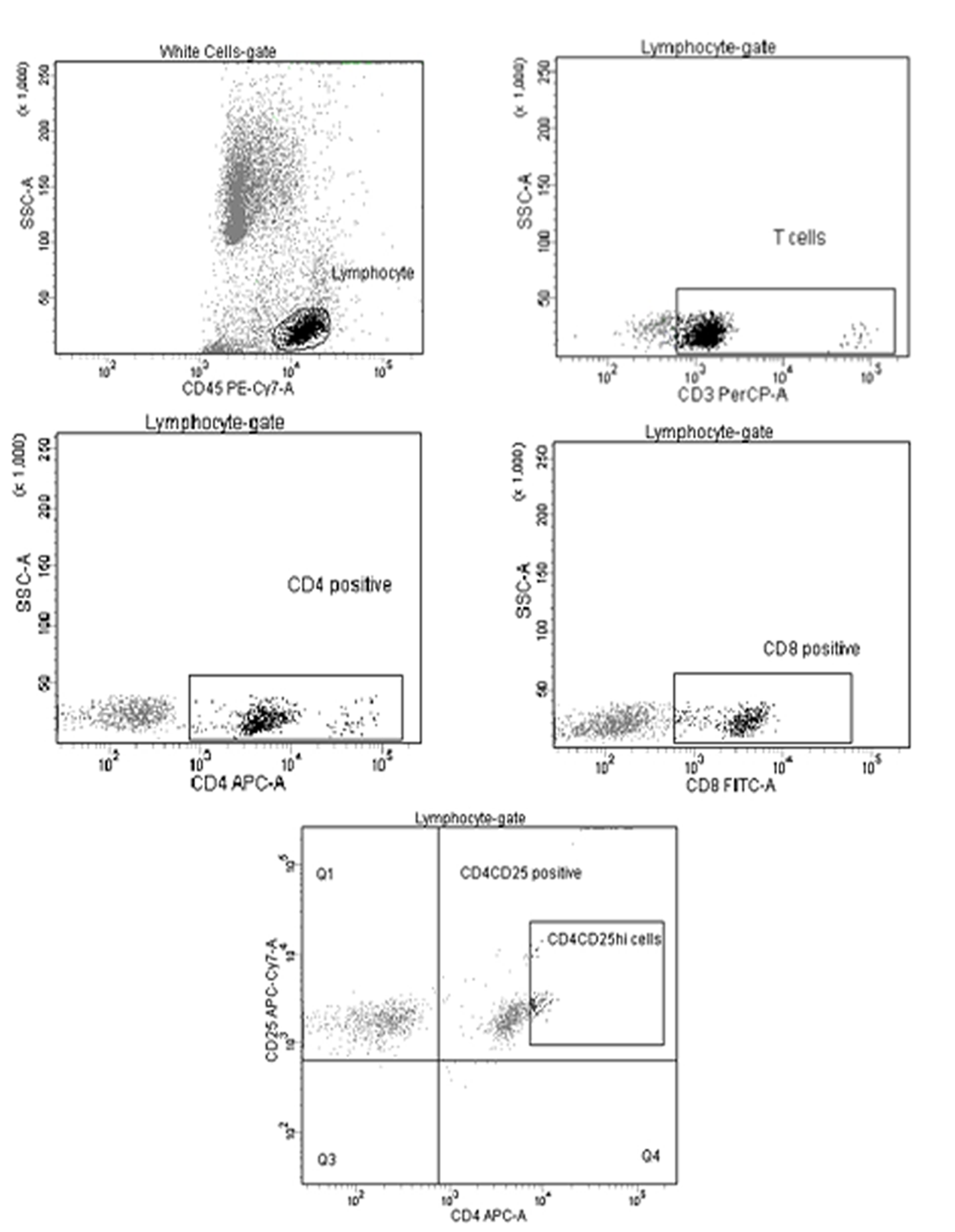

Supplement: S3 Fig — Dot plots shown are representative of one VL patient. Lymphocytes were selected based on low SSC vs. high CD45-PE-Cy7 count and fifty thousand events were acquired. CD45+ gated lymphocytes were further analyzed for CD3, CD4, CD8, CD4CD25 and CD4CD25hi expression. The cells with the phenotype CD4CD25 and CD4CD25hi were considered to be Treg lymphocytes. (TIF) [file pntd.0004422.s003.tif]

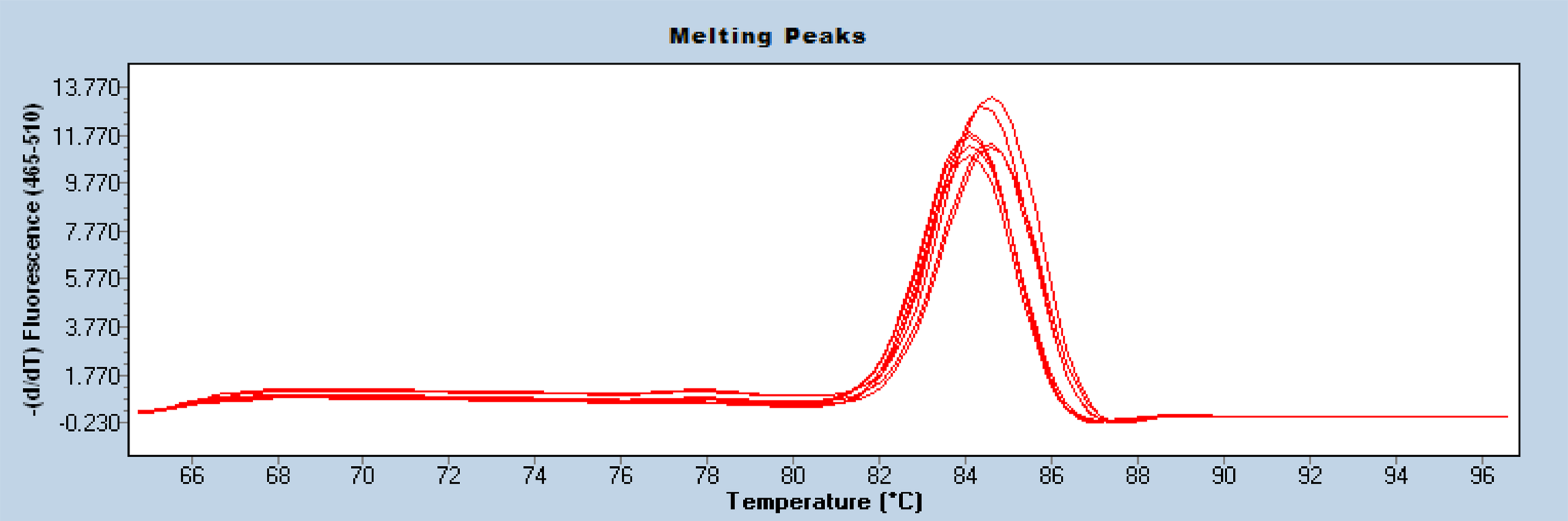

Supplement: S4 Fig — Data shown are for four sets of patient samples. Peaks of curves indicate the melting temperature of the amplicon. (TIF) [file pntd.0004422.s004.tif]

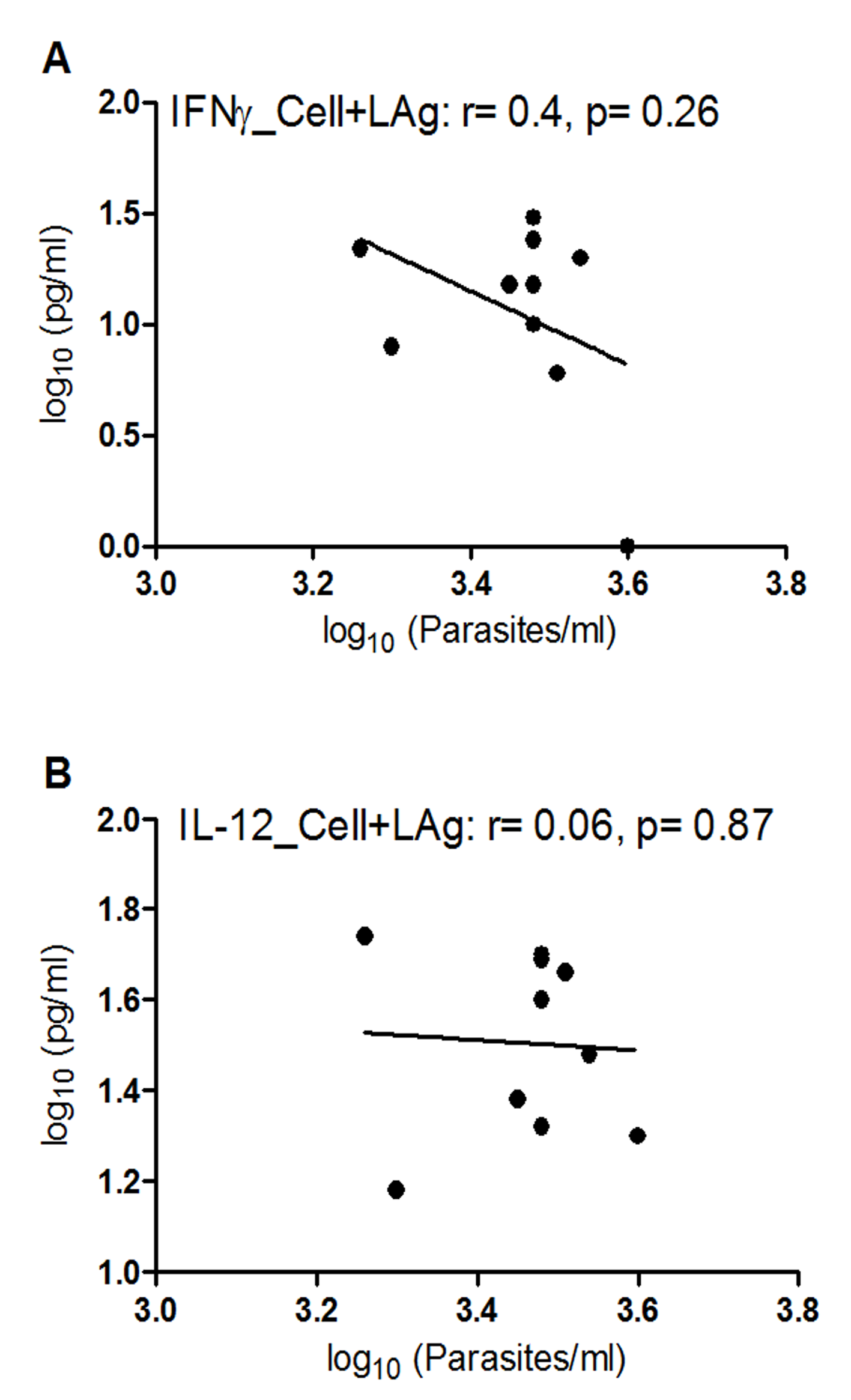

Supplement: S5 Fig — The LAg-stimulated levels (pg/ml) of (A) IFNγ and (B) IL-12 in PBMCs of VL patients were measured by ELISA, and parasite loads (Parasites/ml) were determined by real-time PCR. Correlation was calculated using Spearman/Pearson correlation test. Diagonal lines represent linear regression. (TIF) [file pntd.0004422.s005.tif]

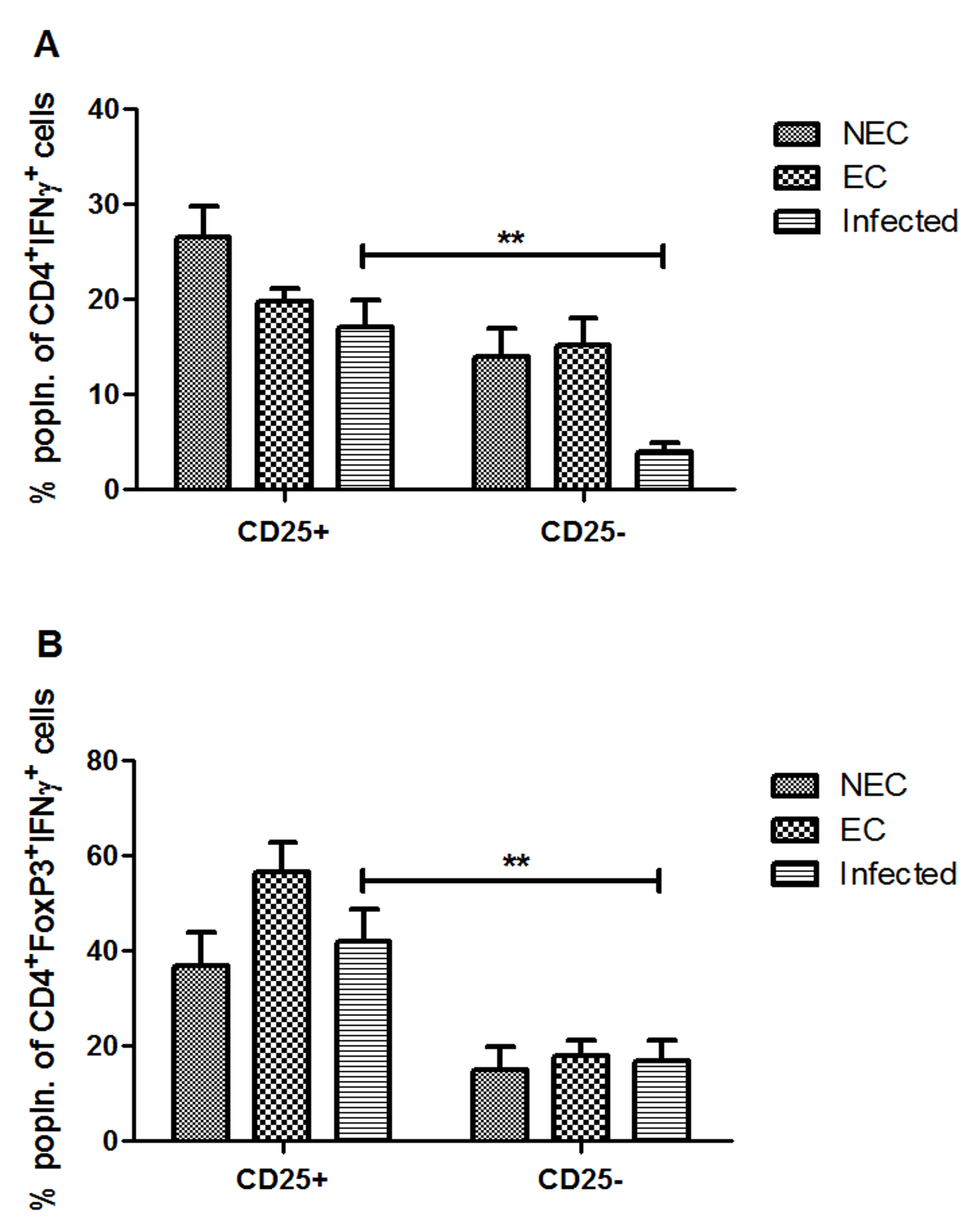

Supplement: S6 Fig — Total PBMCs were freshly cultured in the presence of PMA (50 ng/μl), ionomycin (1 μg/μl) for 2 hrs and for additional 1 hr in presence of brefeldin A (10 μg/μl) before staining. (A) Percentages of CD25+ and CD25− cells among CD4+ IFNγ+ cells. (B) Percentages of CD25+ and CD25− cells among CD4+FoxP3+ IFNγ+ cells. Data are represented as mean ± SE. P values were calculated using Wilcoxon matched pairs signed rank test for paired samples; P<0.05 was considered significant. (TIF) [file pntd.0004422.s006.tif]
