# Supplementary material for: Induction of IL-10 and TGFβ from CD4+CD25+FoxP3+ T Cells Correlates with Parasite Load in Indian Kala-azar Patients Infected with Leishmania donovani
Source: PLoS Negl Trop Dis. 2016 Feb 1;10(2):e0004422. doi: 10.1371/journal.pntd.0004422 (PMC4735109; doi:10.1371/journal.pntd.0004422)
Supplement: S1 Table — (DOC) [file pntd.0004422.s007.doc]

**S1 Table. Numerical representation of parasite load and different plasma cytokine profiles of VL patients (n=20).**

| **IFNγ** | | **IL-12** | | **IL-10** | | **TGFβ** | | **IL-17** | | **IL-22** | |
| --- | --- | --- | --- | --- | --- | --- | --- | --- | --- | --- | --- |
| **Parasite load/ml of blood** | **Cytokine levels (pg/ml)** | **Parasite load/ml of blood** | **Cytokine levels (pg/ml)** | **Parasite load/ml of blood** | **Cytokine levels (pg/ml)** | **Parasite load/ml of blood** | **Cytokine levels (pg/ml)** | **Parasite load/ml of blood** | **Cytokine levels (pg/ml)** | **Parasite load/ml of blood** | **Cytokine levels (pg/ml)** |
| 1800 | 68 | 1750 | 80 | 1800 | 58 | 1750 | 230 | 1750 | 6.3 | 2000 | 270 |
| 2000 | 31.5 | 3000 | 190 | 2000 | 130 | 3000 | 340 | 3000 | 4.5 | 3050 | 255 |
| 1500 | 112 | 1000 | 79.5 | 1500 | 70 | 3000 | 344 | 3000 | 3.4 | 3000 | 330 |
| 1750 | 43 | 3000 | 534 | 1750 | 100 | 3000 | 281 | 4500 | 2.5 | 4500 | 370 |
| 3000 | 105 | 3050 | 720 | 3000 | 60 | 4500 | 380 | 795 | 2.7 | 795 | 1050 |
| 3000 | 15 | 4500 | 95 | 3000 | 148 | 795 | 240 | 2400 | 4.3 | 2175 | 245 |
| 3000 | 54 | 795 | 870 | 3000 | 190 | 2175 | 208 | 487.5 | 2.1 | 487.5 | 270 |
| 4500 | 23 | 2175 | 160 | 4500 | 350 | 487.5 | 208 | 1000 | 6.0 | 1000 | 330 |
| 795 | 13.6 | 487.5 | 750 | 2175 | 130 | 1000 | 270 | 5000 | 20 | 5000 | 500 |
| 2175 | 46 | 1000 | 1100 | 1000 | 87 | 5000 | 309 | 5000 | 4.8 | 3400 | 255 |
| 487.5 | 48 | 1000 | 79.5 | 5000 | 140 | 3400 | 360 | 3400 | 4.7 | 3500 | 125 |
| 1000 | 15.8 | 5000 | 175 | 3400 | 44 | 1800 | 470 | 3500 | 6.75 | 1800 | 270 |
| 1000 | 795 | 3000 | 175 | 3500 | 94 | 2000 | 270 | 1800 | 3.6 | 2000 | 310 |
| 5000 | 39 | 1800 | 200 | 3000 | 70 | 1500 | 355 | 2000 | 4.2 | 1500 | 390 |
| 3400 | 14.5 | 2000 | 175 | 1800 | 32.5 | 3000 | 365 | 1500 | 2.1 | 3000 | 125 |
| 3500 | 27 | 1500 | 375 | 2000 | 31 | 2400 | 375 | 3000 | 5.8 | 1800 | 255 |
| 3000 | 34 | 2000 | 220 | 2400 | 78 | 3500 | 410 | 1800 | 2.7 | 2400 | 500 |
| 1800 | 43 | 2000 | 140 | 1600 | 40 | 3000 | 500 | 2000 | 3.3 | 3000 | 330 |
