# Supplementary material for: Induction of IL-10 and TGFβ from CD4+CD25+FoxP3+ T Cells Correlates with Parasite Load in Indian Kala-azar Patients Infected with Leishmania donovani
Source: PLoS Negl Trop Dis. 2016 Feb 1;10(2):e0004422. doi: 10.1371/journal.pntd.0004422 (PMC4735109; doi:10.1371/journal.pntd.0004422)
Supplement: S1 Checklist — (DOC) [file pntd.0004422.s008.doc]

STROBE Statement—checklist of items that should be included in reports of observational studies

|  | | Item No | Recommendation |
| --- | --- | --- | --- |
| **Title and abstract** | | 1 | (*a*) “Induction of IL-10 and TGFβ from CD4^+^CD25^+^FoxP3^+^ T Cells Correlates with Parasite Load in Indian Kala-azar Patients Infected with *Leishmania donovani*: a cross-sectional study” |
|  |  |  | (*b*) **Background:** Visceral leishmaniasis (VL) is distinguished by a complex interplay of immune response and parasite multiplication inside host cells. However, the direct association between different immunological correlates with parasite number remains largely unknown.  **Objective:** To understand how the different immune correlates (cytokines and cellular subsets) influence parasite dynamics during disease.  **Design:** Active VL patient based cross-sectional study.  **Setting:** VL patients admitted to STM, Kolkata and RMRIMS, Patna.  **Methodology/ Principal Findings:** We examined the plasma levels of different disease promoting/protective as well as Th17 cytokines and found IL-10, TGFβ and IL-17 to be significantly correlated (r= 0.52, 0.53 and 0.51 for IL-10, TGFβ and IL-17, respectively) with parasite load in VL patients. We then extended our investigation to a more antigen-specific response and found leishmanial antigen stimulated levels of both IL-10 and TGFβ to be significantly associated (r= 0.71 and 0.72 for IL-10 and TGFβ respectively) with parasite load. In addition to cytokines we also looked for different cellular subtypes that could attribute to cytokine secretion and parasite persistence. Our observations manifested an association between different Treg cell markers with disease progression as absolute numbers of CD4^+^CD25^+^ (r= 0.55), CD4^+^CD25^hi^ (r= 0.61) as well as percentages of CD4^+^CD25^+^FoxP3^+^ T cells (r= 0.68) all correlated well with parasite load. Encouraged by these results we attempted a link between these immunological components and interestingly found both CD4^+^CD25^+^ and CD4^+^CD25^+^FoxP3^+^ Treg cells to secrete significantly (*p*˂0.05) higher amounts of not only IL-10 but also TGFβ in comparison to corresponding CD25^-^ T cells.  **Conclusions/Significance:** Our findings, for the first time, shed some lights on source(s) of TGFβ and suggest an association between these disease promoting cytokines and Treg cells with parasite load during active disease. Moreover, the direct evidence of CD4^+^CD25^+^FoxP3^+^ Treg cells as a source of IL-10 and TGFβ during active VL could open new avenues for immunotherapy towards cure of this potentially fatal disease. |
| Introduction | | | |
| Background/rationale | | 2 | Visceral leishmaniasis (VL) is one of the most widespread parasitic diseases worldwide caused by the kinetoplastid protozoan of *Leishmania donovani* complex. The disease begins with internalization of *L. donovani* parasites and their multiplication within host macrophages followed subsequently with immune suppression. However, the immunological factors responsible for disease progression and their association with parasite dynamics are not completely understood. Herein, we investigated the correlation of different immune components (cytokines and cellular subsets) with parasite load to predict their involvement in the course of VL. |
| Objectives | | 3 | Our primary objective was to to understand how the immune response generated during VL in the host is impacted by the multiplication of the pathogen resulting in clinical pathogenesis and disease. We would further like to investigate the association between different immune correlates by assigning cellular sources to different disease promoting cytokines. |
| Methods | | | |
| Study design | | 4 | Present study is a cross-sectional study involving different immunological attributes and their influence on parasite progression during active VL. Patients included were from two different regions (Kolkata and Patna) hospitalized during the period of May, 2013 to may, 2014. We analyzed the association of parasite load with circulating and antigen-specific levels of different cytokines along with different T cell subsets. From these outcomes we have tried to predict the sources of different cytokines during disease. |
| Setting | | 5 | Twenty VL patients (males and females, and HIV negative) admitted to School of Tropical Medicine (STM), Kolkata, West Bengal and Rajendra Memorial Research Institute of Medical Sciences (RMRIMS), Patna, Bihar during the period of May, 2013 to May, 2014 were included in this study. Control subjects (both males and females) from endemic and non-endemic regions were also included in the study. |
| Participants | | 6 | (*a*) Patients were initially diagnosed by the usual clinical presentations such as prolonged fever, hepatosplenomegaly and pancytopenia. Confirmation criteria for the disease, however, was restricted to rK39 strip test as risky and painful nature of aspiration (both splenic and bone marrow) techniques limit them to be employed for all patients studied. The non-endemic healthy individuals (NEC), both male and female, included in the study were from different areas of Kolkata with age range of 23-35 years. Endemic healthy individuals (EC) [healthy family members living in the same endemic regions of Bihar] chosen from among the persons attending patients, both males and females with age range of 35-50 years, were also included in the study. |
|  |  |  | (*b*) *Cohort study*— Not applicable  *Case-control study*— Not applicable |
| Variables | | 7 | Only VL patients and no HIV-VL co-infected cases were included in this study. Patients were initially diagnosed by the usual clinical presentations such as prolonged fever, hepatosplenomegaly and pancytopenia. Confirmation criteria for the disease, however, was restricted to rK39 strip test only. |
| Data sources/ measurement | | 8* | Blood samples from VL patients and controls of two different regions were prepared and analyzed using same protocol and by same personnel without making any procedural discrimination based on the origin and/or nature of samples. |
| Bias | | 9 | Endemic healthy controls were also included in the study along with non-endemic controls to counter any bias originating from endemicity of control samples. |
| Study size | | 10 | The number of VL cases (20) admitted to STM, Kolkata and RMRIMS, Patna during the period of May, 2013 to May, 2014 and from whom we were able to collect blood samples determined the study size. |
| Quantitative variables | | 11 | Cytokines and cellular subsets having correlation coefficient (r) of 0.5 or more than that, as calculated using Spearman/Pearson correlation test, were considered to be strongly correlated with parasite load. Those immune correlates were further investigated in the cellular sources study. |
| Statistical methods | | 12 | Correlation between parasite load and different clinical parameters and immune correlates was calculated using Spearman/Pearson correlation test. Diagonal lines represent linear regression. Cytokine producing abilities of different groups or cellular subsets under different conditions were compared using either Mann-Whitney *U* test or Unpaired t test or Wilcoxon matched pairs signed rank test. In all the cases *P*˂0.05 was considered significant. |
|  |  |  |  |
|  |  |  |  |
|  |  |  | (*d*) *Cohort study*— Not applicable  *Case-control study*— Not applicable  *Cross-sectional study*— Not applicable |
|  |  |  | (*e*) Not applicable |
| Results | | | |
| Participants | 13* | (a) Twenty VL patients (males and females, and HIV negative) admitted to School of Tropical Medicine (STM), Kolkata, West Bengal and Rajendra Memorial Research Institute of Medical Sciences (RMRIMS), Patna, Bihar were included in this study. Correlation between antigen-stimulated cytokines and parasite load as well investigation on cytokine sources, however, could only be carried out in 10 VL patients due to requirement of minimum of 4 × 10^6^ cell count in these studies. | |
|  |  | (b) Not applicable | |
|  |  | (c) Not applicable | |
| Descriptive data | 14* | (a) Description of the study population by age, clinical and haematological parameters (Mean ± SD).   | **Characteristics** | **VL patients (n=20)** | | --- | --- | | Age (yrs) | 32.31 ± 16.99 | | Sex  Male 14  Female 6 | | | Body weight (Kg) | 45.90 ± 13.86 | | Spleen size (cm) | 8.04 ± 2.76 | | Haemoglobin (g/dl) | 7.95 ± 1.47 | | WBC count (cells/µl) | 2840 ± 650 | | Albumin (gm/dl) | 3.28 ± 0.71 | | Globulin (gm/dl) | 4.897 ± 0.32 | | Albumin/Globulin | 0.67 | | Platelet count (×10^3^) | 142 ± 1.48 | | Albumin (gm/dl) | 3.28 ± 0.71 | | Parasite load (parasites/ml) | 2549 ± 1213 | | |
|  |  | (b) Not applicable | |
|  |  | (c) Not applicable | |
| Outcome data | 15* | *Cohort study*— Not applicable | |
|  |  | *Case-control study—* Not applicable | |
|  |  | *Cross-sectional study—* Results of *Leishmania* DNA quantification in blood samples and clinical parameters of VL patients.   \| **Patient nos.** \| **Parasite load/ ml of blood** \| **Duration of illness (mo)** \| **Spleen size (cm)** \| **WBC**  **(cells/µl)** \| **Albumin**  **(gm/dl)** \| \| --- \| --- \| --- \| --- \| --- \| --- \| \| **1.** \| 1820 \| 3.0 \| 6.0 \| 1500 \| 3.78 \| \| **2.** \| 1995 \| 1.5 \| 10.0 \| 1100 \| 3.30 \| \| **3.** \| 1514 \| 2.0 \| 3.0 \| 5000 \| 3.70 \| \| **4.** \| 1738 \| 1.0 \| 10.0 \| 2800 \| 3.80 \| \| **5.** \| 3020 \| 3.0 \| 10.0 \| 4800 \| 3.22 \| \| **6.** \| 3020 \| 1.0 \| 7.5 \| 4100 \| 3.70 \| \| **7.** \| 3020 \| 6.0 \| 7.5 \| 3700 \| 3.04 \| \| **8.** \| 4467 \| 6.0 \| 8.0 \| 1500 \| 3.90 \| \| **9.** \| 794 \| 1.0 \| 5.0 \| 4800 \| 3.80 \| \| **10.** \| 2188 \| 2.0 \| 6.0 \| 4000 \| 3.25 \| \| **11.** \| 487.5 \| 3.0 \| 3.0 \| 5200 \| 4.22 \| \| **12.** \| 1000 \| 2.0 \| 10.0 \| 3700 \| 3.92 \| \| **13.** \| 3981 \| 12.0 \| 12.0 \| 2000 \| 3.24 \| \| **14.** \| 5000 \| 12.0 \| 12.0 \| 1000 \| 1.40 \| \| **15.** \| 3388 \| 3.0 \| 8.0 \| 2700 \| 3.70 \| \| **16.** \| 3467 \| 12.0 \| 7.0 \| 2600 \| 2.80 \| \| **17.** \| 3020 \| 2.0 \| 8.0 \| 3020 \| 2.40 \| \| **18.** \| 1820 \| 1.0 \| 6.0 \| 2700 \| 2.50 \| \| **19.** \| 1995 \| 1.0 \| 9.0 \| 2100 \| 3.70 \| \| **20.** \| 3236 \| 8.0 \| 10.0 \| 2000 \| 2.20 \| | |
| Main results | 16 | (*a*) Our study revealed a significant positive correlation between parasite load and plasma as well as antigen specific levels of IL-10 and TGFβ. In addition to cytokines, cellular subsets could also contribute in disease pathogenesis through their regulatory mechanisms. Our results indicate different Treg cell markers (absolute numbers of CD4^+^CD25^+^ and CD4^+^CD25^hi^ and percentages of CD4^+^CD25^+^FoxP3^+^) to be strongly correlated with parasite load. Exploring an association between these immunological correlates revealed Treg cells to be the source of these cytokines during VL. | |
|  |  | (*b*) Not applicable | |
|  |  | (*c*) Not applicable | |
| Other analyses | 17 | Not applicable | |
| Discussion | | | |
| Key results | 18 | Our study, in addition to IL-10, for the first time demonstrates a positive correlation of TGFβ in plasma as well as IL-10 and TGFβ in antigen-stimulated PBMCs from culture supernatants with parasite load of VL patients supporting their roles in disease pathology. Additionally, our results also point to a positive correlation between absolute numbers of CD4^+^CD25^+^ Treg cells with parasite load, highlighting their involvement in *L. donovani* infection. On the basis of the initial results we further investigated whether these cellular subsets could be attributed as the sources of disease promoting cytokines (IL-10 and TGFβ) and our observation, along with IL-10, for the first time revealed TGFβ to be secreted from CD4^+^CD25^+^FoxP3^+^ Treg cells as well in during active VL. | |
| Limitations | 19 | Principally our study aimed to investigate how multiplication of parasites was influenced by different immune components during active VL. To carry out this study we have quantified parasites present in blood of VL patients. Although, since this disease is marked by visceral infection, it would be more appropriate if the parasite load could be determined in infected organs (like spleen and bone marrow) rather than circulation. However, considering the pain and fatal risk involved in extracting parasites from these organs, it is not a common practice. | |
| Interpretation | 20 | Collectively our data suggest a possible role of Tregs, IL-10 and TGFβ in parasite establishment and course of disease progression among VL patients. The finding that CD4^+^CD25^+^FoxP3^+^ Treg cells could produce both IL-10 and TGFβ upon *L. donovani* infection is of prime importance in the knowledge of VL pathogenesis. Such findings could lead us to devise new vaccine strategies or immunotherapies against VL, important from the perception of eradication of parasites and cure of VL. | |
| Generalisability | 21 | Reports regarding the involvement of regulatory T cells (Tregs) in active VL and their secretory role of disease promoting cytokines are few and inconclusive. We tried to present a general picture by including patients from two geographical regions encompassing two states (Bihar and West Bengal) endemic for VL. While the size of our study group was on the lower side (n=20), the range was big: male and female, variable age groups and patients with different parasite load were well represented. Moreover, we also included asymptomatic healthy individuals from both endemic and non-endemic regions as controls in our study. | |
| Other information | | | |
| Funding | 22 | Council of Scientific and Industrial Research (CSIR), India and Indian Council of Medical Research (ICMR), India. PB is a Senior Research Fellow of ICMR. | |

*Give information separately for cases and controls in case-control studies and, if applicable, for exposed and unexposed groups in cohort and cross-sectional studies.

**Note:** An Explanation and Elaboration article discusses each checklist item and gives methodological background and published examples of transparent reporting. The STROBE checklist is best used in conjunction with this article (freely available on the Web sites of PLoS Medicine at http://www.plosmedicine.org/, Annals of Internal Medicine at http://www.annals.org/, and Epidemiology at http://www.epidem.com/). Information on the STROBE Initiative is available at www.strobe-statement.org.
